# Nonfatal Burden of Disease and Risk-Attributable Burden in Romania, 1990 to 2023: A Secondary Analysis of Global Burden of Disease 2023 Estimates

**DOI:** 10.3390/healthcare14183035

**Published:** 2026-09-16

**Authors:** Mihaela Hostiuc, Vlad-Adrian Afrasanie, Octavian Andronic, Alina-Ioana Forray, Sorin Hostiuc, Andreea-Iuliana Ionescu, Radu-Tudor Ionescu, Paschalis Karakasis, Ana Maria Musina, Ruxandra Negoi, Bogdan Oancea, Dimitrios Patoulias, Mugurel Constantin Rusu, Alexandru Scafa, Ionut Negoi

**Affiliations:** 1 Department of Internal Medicine, Carol Davila University of Medicine and Pharmacy, 050474 Bucharest, Romania; mihaela.hostiuc@umfcd.ro; 2 Department of Oncology, Grigore T. Popa University of Medicine and Pharmacy, 700115 Iași, Romania; vlad_afrasanie@yahoo.com (V.-A.A.); musina.anamaria@gmail.com (A.M.M.); 3 Regional Institute of Oncology, 700483 Iași, Romania; 4 Department of Surgery, Carol Davila University of Medicine and Pharmacy, 050474 Bucharest, Romania; octavian.andronic@umfcd.ro (O.A.); ionut.negoi@umfcd.ro (I.N.); 5 Department of Community Medicine, Faculty of Medicine, Iuliu Hațieganu University of Medicine and Pharmacy, 400347 Cluj-Napoca, Romania; alina.forray@umfcluj.ro; 6 Department of Legal Medicine and Bioethics, Carol Davila University of Medicine and Pharmacy, 050474 Bucharest, Romania; 7 National Institute of Legal Medicine Mina Minovici, 077160 Bucharest, Romania; 8 Department of Medical Oncology, Carol Davila University of Medicine and Pharmacy, 050474 Bucharest, Romania; andreea-iuliana.ionescu@umfcd.ro; 9 Faculty of Mathematics and Computer Science, University of Bucharest, 030018 Bucharest, Romania; radu.ionescu@securif.ai; 10 Second Department of Cardiology, Hippokration General Hospital, Aristotle University of Thessaloniki, 541 24 Thessaloniki, Greece; pkarakaa@auth.gr; 11 Department of Cardiology, Carol Davila University of Medicine and Pharmacy, 050474 Bucharest, Romania; negoiruxandra@gmail.com (R.N.); alexandru.scafa@umfcd.ro (A.S.); 12 Department of Computer Science, University of Bucharest, 030018 Bucharest, Romania; bogdan.oancea@faa.unibuc.ro; 13 National Institute for Research and Development in Biological Sciences, 060031 Bucharest, Romania; 14 Second Propedeutic Department of Internal Medicine, Aristotle University of Thessaloniki, 541 24 Thessaloniki, Greece; patoulias@auth.gr; 15 Department of Anatomy, Carol Davila University of Medicine and Pharmacy, 050474 Bucharest, Romania; mugurel.rusu@umfcd.ro

**Keywords:** global burden of disease, years lived with disability, healthy life expectancy, risk factors, population ageing, noncommunicable diseases, Romania, Central Europe

## Abstract

**Background/Objectives:** We assessed changes in nonfatal disease burden and risk attribution in Romania between 1990 and 2023. **Methods:** We analysed Global Burden of Disease (GBD) 2023 estimates of years lived with disability (YLDs), related nonfatal measures and risk attribution for 199 causes and 61 risks, with seventeen comparator locations. **Results:** The age-standardised YLD rate changed by −4.1% (95% uncertainty interval [UI] −10.1 to 1.3), and total YLDs by −3.9% (95% UI −9.2 to 0.7). Both intervals included zero. The crude rate rose by 17.0%. The population size and age structure components of the decomposition were approximately −552,000 and +545,000 YLDs; their uncertainty was not estimated. Healthy life expectancy increased by 5.2 years. Its difference from life expectancy rose from 9.2 to 10.2 years, without an uncertainty interval for this derived change. Low-back pain remained the leading level 3 cause. Anxiety disorders increased by 41.7% (95% UI 4.5 to 101.8) and ranked second by point estimate; rank uncertainty was not estimated. The joint risk-attributable fraction fell from 31.2% to 28.3%. **Conclusions:** Romania had more disability per inhabitant and a larger share at older ages in 2023. Musculoskeletal disorders remained the main source of nonfatal burden, while anxiety disorders and diabetes increased.

## 1. Introduction

Years lived with disability (YLDs) capture the non-fatal component of disease burden by combining the prevalence of each sequela with the severity of the health loss it causes, complementing years of life lost (YLLs), which are derived from premature mortality, within the broader disability-adjusted life-year (DALY) framework [1,2]. Earlier GBD analyses documented the distribution of nonfatal health loss across diseases, ages and countries [3,4]. Changes in population size and age structure affect disability counts, while changes in disease occurrence, duration and survival affect age-specific rates [1,5,6]. The demand for long-term treatment, rehabilitation, mental health services and care comes from conditions that kill rarely, led by musculoskeletal disorders, headache disorders and mental disorders.

Across the 27 member states of the European Union, age-standardised death and years of life lost rates fell between 2010 and 2019, while rates of years lived with disability stayed near their 2010 values, reproducing the same split between the two components; diabetes and kidney diseases were the cause group whose age-standardised disability-adjusted rate rose, by 3.5% [7].

Work on east–west health differences in Europe has focused mainly on mortality [8,9]. Earlier cross-country work on injury inequalities in Europe relied mainly on mortality rate ratios and left aside measures that combine fatal and non-fatal outcomes [10]. In 2019, injury mortality in Eastern Europe was 80 per 100,000, compared with 38 in Central Europe and 27 in Western Europe [10]. The corresponding injury DALY rates were 5129, 2940 and 1782 per 100,000, with ratios of similar sizes on both measures; the burden measure adds the absolute scale of the loss, with several thousand years per 100,000 separating east from west once nonfatal outcomes are counted [10].

For Romania, we have undertaken a single national analysis of non-fatal health loss using Global Burden of Disease estimates, focused on mental and substance use disorders, which ranked third among causes of non-fatal burden in 2016 and accounted for 13.5% of total years lived with disability [11]. That same analysis found that mental and substance use disorders were the fifth leading contributor to total disease burden, accounting for 5.5% of DALYs, and that depression, anxiety, and alcohol use disorders carried the highest burden within this cause group [11].

The rest of the Romanian evidence base consists of condition-specific national surveys, and no published study has mapped morbidity across the full GBD cause hierarchy. The Romanian subgroup of the Rome Foundation Global Epidemiology Study included 2049 respondents with good national geographic representation, and found that 40.1% met criteria for at least one disorder of gut–brain interaction [12]. The PREDATORR study, a cross-sectional national survey of 2717 adults, estimated the age- and sex-adjusted prevalence of chronic kidney disease at 6.7% and identified older age, hyperuricaemia, impaired glucose regulation, hypertriglyceridaemia, and family history of renal disease as independent correlates [13].

A more recent population health survey from Romania showed marked rural–urban inequalities in risk-factor profiles and access to care: rural residents had lower education and income, higher binge drinking, more high blood pressure, more overweight and obesity, and higher fasting plasma glucose than urban residents [14]. Urban residents showed higher proportions of smoking, daily alcohol consumption, and hypercholesterolaemia [14].

The primary aim was to characterise trends in Romania’s non-fatal disease burden between 1990 and 2023, and to compare Romania with Central, Eastern and Western Europe and with neighbouring countries. The secondary aim was to identify the causes and attributable risks whose estimates changed over the period.

## 2. Materials and Methods

### 2.1. Study Design and Data Source

We used modelled estimates extracted from the GBD Results tool of the Institute for Health Metrics and Evaluation for the years 1990 to 2023. The latest source exports were downloaded on 4 August 2026. The estimation framework, the input data, the models and the uncertainty propagation are described in the study capstone [1] and in its methods appendices, and are not reproduced here. The study follows the Guidelines for Accurate and Transparent Health Estimates Reporting [15]; the completed checklist appears in Table S1.

Population figures used as denominators and for the decomposition come from the same source, which draws them from the GBD demographic estimates [16].

We included estimates for 1990–2023 in Romania and the comparator locations listed in Section 2.4, using the measures and hierarchy levels specified in Section 2.2 and Section 2.3. Male, female and both-sex estimates were retained. National totals and crude rates used all ages; age-standardised rates used the GBD standard population. Age-specific analyses used twenty groups, from under 5 years to 95 years and older. HALE and life expectancy were analysed at birth and at ages 65–69 years where available. We excluded records outside the selected years and locations, fatal measures as outcomes, level 4 risks and the five overlapping cause aggregates specified below. Missing categories were excluded; explicit zero estimates were retained. Disability-adjusted life-years were used only as denominators for the nonfatal share of total burden.

### 2.2. Measures

The analysis is restricted to nonfatal burden and to burden attributable to risk factors expressed in nonfatal terms. It reports years lived with disability, prevalence, incidence, healthy life expectancy, the summary exposure value and population attributable fractions computed on years lived with disability. Deaths and years of life lost fall outside its scope, and are reported for Romania in a companion analysis of causes of death; disability-adjusted life-years enter only as a denominator, in the comparison between the nonfatal and the total burden of a cause.

Years lived with disability are the product of prevalence and a disability weight, summed over the sequelae of a cause and corrected for comorbidity, so that the sum across causes does not exceed the burden a person can carry. Healthy life expectancy at birth is derived from the life table and the age-specific prevalence of disability by the Sullivan method [17]. The summary exposure value expresses the exposure of a population to a risk on a scale from zero to one, relative to the exposure that would produce the maximum burden, and is reported here as a percentage. All these quantities, together with the comparative risk assessment framework, the theoretical minimum risk exposure level and the mediation of risk effects, follow the definitions of the capstone cited above.

Rates are age-standardised to the GBD world standard population unless stated otherwise. Uncertainty intervals are the 95% intervals reported by the source, bounded by the 2.5th and 97.5th percentiles of the 250 draws that GBD 2023 propagates through every step of the estimation; the propagation was performed within GBD, and the present analysis reuses the published summaries. Population figures and derived shares carry no uncertainty interval in the source and are reported as point estimates.

### 2.3. Cause and Risk Hierarchies

The cause hierarchy of GBD 2023 has 3 top-level groups, 22 groups at level 2 and 176 at level 3. The results tool serves 174 of the level 3 causes, so the analysis covers 199 causes across the three levels plus the all-cause aggregate. Five further categories that the hierarchy tables label as level 1, namely, total cancers, total cancers excluding non-melanoma skin cancer, and the totals related to hepatitis B, hepatitis C and non-alcoholic fatty liver disease, aggregate across the hierarchy and were excluded, since retaining them would count the same cases twice in any ranking.

The YLD export contained 200 categories including the all-cause aggregate. Availability differed by measure; the prevalence export contained 197 categories including all causes, and the incidence export contained 184. Prevalence was unavailable for other chronic respiratory diseases, other intestinal infectious diseases and other nutritional deficiencies. Incidence was unavailable for these three and for age-related and other hearing loss, blindness and vision loss, cysticercosis, food-borne trematodiases, hypertensive heart disease, idiopathic developmental intellectual disability, intestinal nematode infections, lymphatic filariasis, onchocerciasis, other cardiovascular and circulatory diseases, other musculoskeletal disorders, schistosomiasis and trachoma. Each analysis used the categories actually returned for its measure, without imputing the absent estimates.

The risk hierarchy has 3 groups at level 1, 20 at level 2 and 42 at level 3. The tool returned estimates for 61 of these 65 risks for Romania; non-optimal temperature with its two components and ambient ozone pollution carry an exposure estimate without an attributable nonfatal burden. Level 4 of the risk hierarchy, with 22 positions, falls outside the analysis.

### 2.4. Comparator Locations

Romania is compared with the twelve other countries of the GBD Central Europe region, namely, Albania, Bosnia and Herzegovina, Bulgaria, Croatia, Czechia, Hungary, Montenegro, North Macedonia, Poland, Serbia, Slovakia and Slovenia, and with four aggregates, the Central Europe, Eastern Europe and Western Europe regions, and the global estimate. The Central Europe, Eastern Europe and Central Asia super-region is reported alongside them, giving eighteen locations in all. Regional membership follows the GBD location hierarchy [18]. The Eastern Europe region enters the comparison because the post-Soviet states followed a different transition path from the Central European ones, and Western Europe because it marks the upper bound of health attainment in the wider region.

### 2.5. Time Window

The window runs from 1990 to 2023 and every year is retained. Prevalence, incidence, years lived with disability and risk-attributable burden begin in 1990 in GBD 2023, unlike deaths and years of life lost, which begin in 1980.

### 2.6. Analysis

Point estimates and their uncertainty intervals are reported as extracted. Change between 1990 and 2023 is reported as the percentage change with its own 95% uncertainty interval, obtained from the results tool by the same draw-level computation that produces the levels, so that the uncertainty of the difference comes from the draws themselves and no inference from the two annual intervals is needed. A change was classified as increasing or decreasing when the 95% uncertainty interval for percentage change excluded zero, and as directionally uncertain when it included zero; this terminology describes estimation uncertainty and does not constitute formal hypothesis testing. The evaluations across the 22 level 2 causes, the 174 level 3 causes and the 61 risks are exploratory and carry no multiplicity adjustment. The cause and risk hierarchies make these comparisons dependent, so the number of intervals excluding zero was not used as a global test. We distinguish changes whose intervals narrowly exclude zero from those with bounds further from zero. Uncertainty in ranks requires separate estimation. Attributable fractions depend on relative risks and on the distribution of burden, as well as on exposure, so a change in a fraction is not read as a proportional change in exposure; exposure trends are carried by the summary exposure value. Comparisons between locations in a single year, for which the tool does not compute a difference, are described through the distance between point estimates, as a percentage of the reference value or as a difference in percentage points, with a statement of whether the two annual intervals overlap; the overlap of two marginal intervals is not a test of the difference between locations, so these comparisons remain descriptive. Ranks were computed on point estimates within each hierarchy level and each year. Countries are ranked separately from regional and global aggregates, rank uncertainty was not quantified, and, because Romania contributes to the Central Europe aggregate, that comparison serves as a part–whole benchmark.

The change in the absolute number of years lived with disability between 1990 and 2023 was decomposed into three components—the size of the population, its age structure and the age-specific rates—following the symmetric approach of Das Gupta [19]. Writing the total as the product of the population and the sum over age groups of the age share and the age-specific rate, the population component is the change in population size weighted by the mean of the two composite rates, and the remainder is split between age structure and rates by weighting each change with the mean of the other factor across the two years. The three components sum exactly to the change in the estimated count. The exports contain point estimates and marginal uncertainty limits for rates and populations. They do not contain the matched draws needed to calculate intervals for the decomposition components or their balance. We report the components to the nearest thousand YLDs in the text and retain the unrounded calculations in Table S2. Applying the extracted age-specific rates to the extracted populations reproduces the national totals to the unit, which serves as an arithmetic check on the procedure. The decomposition was run for both sexes together and separately for men and women.

Analyses were carried out in R 4.6.1 (R Foundation for Statistical Computing, Vienna, Austria) within R Studio 2026.09.0 (Posit Software, PBC, Boston, MA, USA). Data import, reshaping, tabulation and validation used data.table 1.18.4; figures were drawn with base R graphics.

### 2.7. Data Availability and Ethics

All source estimates are publicly available through the GBD Results Tool. The supporting tabulations are included in the Supplementary Materials. The analysis code is available from the corresponding author upon reasonable request.

We used publicly available estimates for national populations. The records contained no names, personal identifiers or individual clinical data. No person-level data were linked or disclosed. Ethical approval and individual consent were not required, as this is an ecological study, not directly involving human subjects.

## 3. Results

### 3.1. Nonfatal Burden of Disease

Romania recorded 2,840,268 years lived with disability (95% uncertainty interval 2,160,171 to 3,645,794) in 1990 and 2,729,113 (2,098,146 to 3,486,352) in 2023. The count changed by −3.9% (95% UI −9.2 to 0.7); the age-standardised rate changed by −4.1% (95% UI −10.1 to 1.3), from 11,196.8 per 100,000 population (8496.6 to 14,392.7) in 1990 to 10,734.5 (8175.8 to 13,950.4) in 2023. The crude rate rose from 120.7 in 1990 to 141.2 years lived with disability per 1000 population in 2023, an increase of 17.0%.

**Figure 1:**
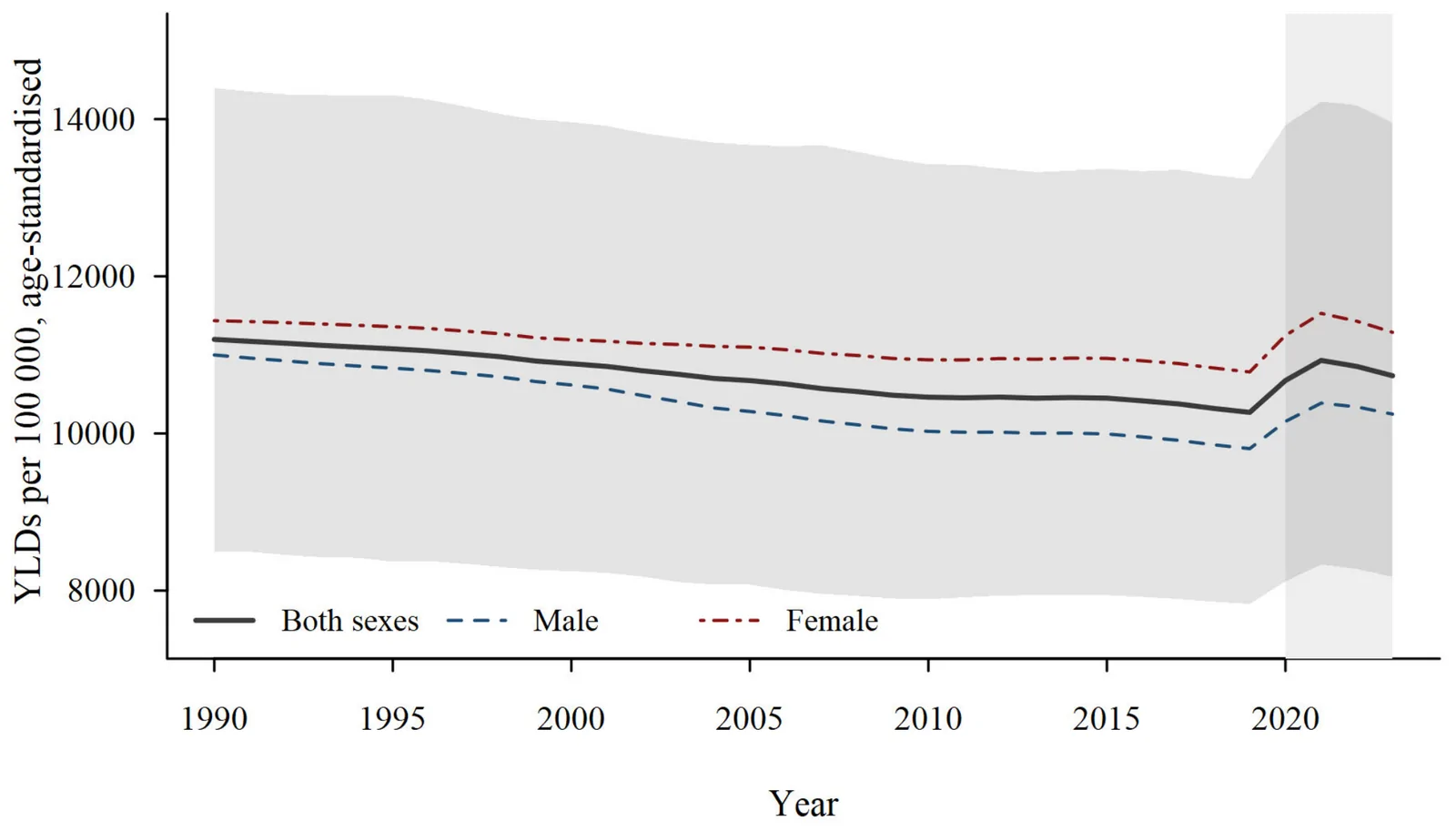
Age-standardised rate of years lived with disability from all causes, Romania, 1990 to 2023. The shaded band around the line for both sexes is the 95% uncertainty interval. The grey panel marks the years 2020 to 2023.

The age-standardised rate declined across every five-year interval up to a series minimum in 2019, then rose through 2020 and 2021 and settled 4.5% above that minimum in 2023, above every annual value recorded between 2004 and 2020 (Figure 1). On point estimates, the change splits into a decrease of 8.3% between 1990 and 2019, from 11,196.8 to 10,268.2 per 100,000, and a pandemic-period increase of 4.5% between 2019 and 2023; draw-level intervals for the two sub-periods were not extracted. The rate fell in men, by 6.8% (2.3 to 11.6), and was directionally uncertain in women, at −1.3% (−8.3 to 4.6); women carried the higher rate in both years, 11,435.2 per 100,000 (8627.9 to 14,753.2) against 10,998.6 (8388.8 to 14,223.0) in 1990 and 11,286.9 (8564.1 to 14,792.5) against 10,245.9 (7850.7 to 13,107.0) in 2023, so the point-estimate female-to-male ratio increased from 1.0 to 1.1. Table S3 gives the annual point-estimate series by sex.

COVID-19 accounted for 193.5 YLDs per 100,000 in 2023, or 1.8% of the all-cause rate. Subtracting it gives 10,541 per 100,000, 5.9% below the 1990 all-cause rate and 2.7% above the 2019 rate. The corresponding changes with COVID-19 included were −4.1% and +4.5%. This subtraction leaves all other cause estimates unchanged and does not recalculate comorbidity. No uncertainty interval was available for the subtracted rate or its change (Table S4).

The eighteen 2023 age-standardised YLD rates ranged from 10,676 to 11,679 per 100,000, with overlapping 95% uncertainty intervals. Romania ranked third-lowest by point estimate among both the eighteen locations and the thirteen Central European countries. These rankings have no uncertainty intervals, and no draw-wise comparisons between locations were available (Table S5). Percentage-change intervals excluded zero for Western Europe, Hungary and Montenegro; they included zero for Romania, Central Europe, Eastern Europe and the global estimate (Table S6).

### 3.2. Leading Causes of Years Lived with Disability

Non-communicable diseases carried 73.3% of the all-cause age-standardised rate in 1990 and 77.7% in 2023, injuries carried 16.7% and 12.6%, and communicable, maternal, neonatal and nutritional diseases carried 9.9% and 9.8%. Of the three groups only injuries had a 95% uncertainty interval for change that excluded zero, falling by 28.0% (24.7 to 32.7); the intervals for non-communicable diseases and the communicable group included zero. The ten largest level 2 causes covered 83.6% of the total in 1990 and 83.1% in 2023.

Musculoskeletal disorders ranked first among the 22 level 2 causes in both years, at 2091.8 per 100,000 (1528.2 to 2866.0) in 1990 and 2080.8 (1543.6 to 2848.9) in 2023, had a change interval that included zero, and represented 19.4% of the all-cause rate in the final year. On point estimates, mental disorders moved from third to second and unintentional injuries from second to third, the first rising by 16.4% (2.4 to 35.6) and the second falling by 25.2% (21.7 to 29.7), so mental disorders passed from 2.7% below unintentional injuries in 1990 to 51.3% above them in 2023. The 2.4% lower bound was close to zero; no uncertainty interval was available for the rank change. Three causes moved three positions or more; diabetes and kidney diseases moved upward from fourteenth to eighth, nutritional deficiencies moved downward from eighth to twelfth, and substance use disorders moved downward from twelfth to fifteenth.

**Figure 2:**
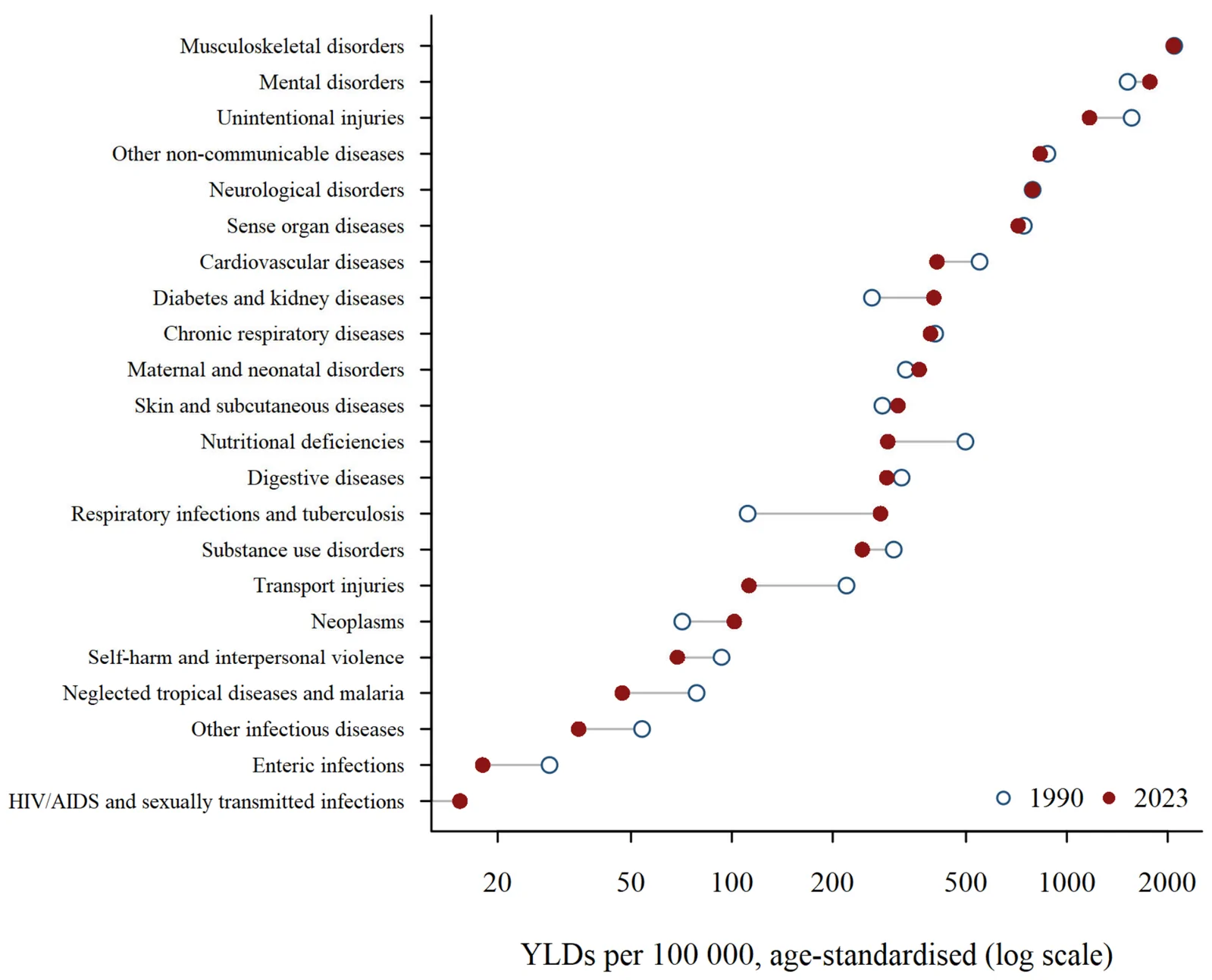
Age-standardised rate of years lived with disability by level 2 cause, Romania, 1990 and 2023, ordered by the 2023 value. The horizontal axis uses a logarithmic scale.

Of the 22 level 2 causes, 14 had 95% uncertainty intervals for change that excluded zero. The six that rose were respiratory infections and tuberculosis by 149.1% (34.3 to 380.7), HIV/AIDS and sexually transmitted infections by 69.4% (39.1 to 133.6), diabetes and kidney diseases by 53.0% (42.0 to 65.4), neoplasms by 43.0% (20.0 to 72.8), mental disorders by 16.4% (2.4 to 35.6), and skin and subcutaneous diseases by 11.3% (7.3 to 15.8). COVID-19, a cause with no 1990 counterpart, carried the rise in respiratory infections and tuberculosis; without it, the four remaining causes in the group summed to 84.3 per 100,000 in 2023 against 111.5 in 1990. The eight that fell were transport injuries, by 48.9% (46.5 to 52.2), enteric infections by 36.8% (30.7 to 42.9), self-harm and interpersonal violence by 26.2% (21.7 to 30.8), cardiovascular diseases by 25.4% (22.0 to 28.8), unintentional injuries by 25.2% (21.7 to 29.7), substance use disorders by 19.4% (8.6 to 29.9), digestive diseases by 9.7% (5.3 to 14.0), and sense organ diseases by 3.8% (0.9 to 6.3). The remaining causes—among them nutritional deficiencies, with a point estimate 41.4% lower, and neglected tropical diseases, with one 40.0% lower—carry intervals that include zero. Figure 2 shows the 22 level 2 causes in both years, Table S7 gives their ranks and intervals and Table S8 gives the percentage changes of each.

Low-back pain ranked first among the 174 level 3 causes in both years, at 1526.3 per 100,000 (1095.6 to 2054.6) in 1990 and 1432.1 (1016.9 to 1930.3) in 2023, and it fell by 6.2% (2.3 to 10.2), while remaining 2.1 times higher than the second-ranked cause. On point estimates, anxiety disorders moved from fifth to second, rising by 41.7% (4.5 to 101.8), and diabetes mellitus recorded the largest upward movement inside the top twenty among causes present in both years, from eighteenth to ninth, rising by 55.0% (43.4 to 67.8). Stroke fell furthest in rank, from twelfth to nineteenth, by 32.1% (27.2 to 36.1). The lower bound for the rate increase was 4.5%, close to zero. No uncertainty interval was available for the rank change. Of the 174 level 3 causes, 95 had change intervals that excluded zero. Table 1 gives the twenty largest, and Table S9 the full list.

Low-back pain headed the level 3 ranking in both sexes in 2023. In men, it was followed by falls, age-related and types of other hearing loss, exposure to mechanical forces and anxiety disorders; in women, it was followed by anxiety disorders, headache disorders, age-related and other hearing loss, and gynaecological diseases. Across the three causes common to both lists, the female rate ran 34.3% above the male one for low-back pain and 89.9% above it for anxiety disorders, while the male rate ran 66.3% above the female one for falls (Table S10).

COVID-19 peaked in 2021 and stood at 193.5 per 100,000 (61.5 to 477.8) in 2023, 36.4% of its peak value and seventeenth amongst the level 3 causes in that year (Table S4).

### 3.3. Change in Cause-Specific Rates Between 1990 and 2023

Of the 174 level 3 causes, 95 had a 95% uncertainty interval for percentage change that excluded zero between 1990 and 2023. Of these 95, 45 rose and 50 fell. Of the 34 causes that reached a rate above 100 per 100,000 in either year, 15 changed, 4 upward and 11 downward, and the remaining 19 had change intervals that included zero.

The four large causes that rose were diabetes mellitus, from 197.8 per 100,000 (134.0 to 270.4) to 306.5 (211.5 to 424.8), an increase of 55.0% (43.4 to 67.8); other musculoskeletal disorders, from 105.3 (67.1 to 154.9) to 160.7 (105.5 to 228.0), an increase of 52.6% (33.5 to 75.8); anxiety disorders, from 486.1 (309.7 to 720.4) to 688.8 (425.3 to 1033.6), an increase of 41.7% (4.5 to 101.8); and osteoarthritis, from 207.0 (97.1 to 456.1) to 228.9 (107.7 to 498.5), an increase of 10.6% (6.9 to 14.0).

The eleven large causes that fell were road injuries, from 199.3 per 100,000 (147.7 to 261.8) to 98.8 (71.1 to 131.9), a decrease of 50.4% (48.3 to 53.6); fire, heat and hot substances, from 129.8 to 65.9, a decrease of 49.2% (40.6 to 58.0); other unintentional injuries, from 160.1 to 87.4, a decrease of 45.4% (42.5 to 48.3); ischaemic heart disease, from 169.6 to 114.3, a decrease of 32.6% (26.2 to 38.3); stroke, from 255.5 to 173.6, a decrease of 32.1% (27.2 to 36.1); exposure to mechanical forces, from 463.0 to 338.7, a decrease of 26.9% (23.2 to 31.4); alcohol use disorders, from 258.9 to 197.4, a decrease of 23.8% (11.8 to 35.0); gallbladder and biliary diseases, from 145.6 to 125.0, a decrease of 14.1% (6.6 to 21.0); blindness and vision loss, from 176.0 to 151.7, a decrease of 13.8% (7.8 to 19.3); falls, from 695.8 to 610.1, a decrease of 12.3% (8.4 to 17.4); and low-back pain, from 1526.3 to 1432.1, a decrease of 6.2% (2.3 to 10.2).

Among the large causes whose change intervals included zero, four carry point estimates within 1% of their 1990 value, namely, age-related and other hearing loss at 551.0 per 100,000 in 2023, headache disorders at 536.1, neck pain at 229.9 and gynaecological diseases at 235.2. Two others carry large point differences with intervals that include zero, namely, dietary iron deficiency, from 477.9 to 286.1 with an interval running from a decrease of 74.6% to an increase of 41.5%, and neonatal disorders, from 324.1 to 356.5 with an interval running from a decrease of 12.4% to an increase of 44.3%.

The remaining 80 causes that changed all lie below 100 per 100,000 in both years. Among those that rose, seven are neoplasms, headed by colon and rectum cancer, from 6.5 per 100,000 (4.6 to 8.7) to 13.4 (9.7 to 17.8), and prostate cancer, from 3.3 to 8.7. HIV/AIDS shows the largest increase in the whole cause list, from 0.8 per 100,000 (0.5 to 1.1) to 7.0 (4.1 to 11.2). Among those that fell, the largest in 1990 were poisonings, from 43.1 per 100,000 to 16.8, tuberculosis, from 33.7 to 12.8, and vitamin A deficiency, from 12.8 to 1.4. Table S11 gives every level 3 cause with its percentage change and interval.

### 3.4. Prevalence and Incidence

Age-standardised prevalence of all causes fell by 2.5% (95% UI 1.8 to 3.2), from 97,553.5 per 100,000 (96,979.6 to 98,060.8) in 1990 to 95,133.7 (94,365.6 to 95,872.5) in 2023, and the case count fell by 18.6% (18.2 to 19.0), from 23.1 to 18.8 million. Age-standardised incidence changed by 19.2% (95% UI −2.7 to 64.1) and was directionally uncertain, moving from 351,343.6 per 100,000 (331,589.4 to 372,560.9) to 418,970.4 (337,390.4 to 586,412.0); the case-count change was 3.2% (95% UI −21.6 to 35.1), also with an interval that included zero. Incidence counts episodes, so one person can contribute several episodes in a year and cause-level rates can sum above 100,000 per 100,000 population.

Respiratory infections and tuberculosis carry the whole of the difference in incidence, rising by 64.1% (9.5 to 176.4) from 142,150 per 100,000 (127,209 to 160,497) to 233,214 (151,738 to 400,643). The group gained 91,065 cases per 100,000 against an all-cause gain of 67,627, so the remaining 21 level 2 causes together carried a lower incidence rate in 2023. Within the group, upper respiratory infections stood within 1% of their 1990 value. COVID-19, a cause absent in 1990, supplied 93,588 per 100,000 (16,460 to 258,049) in 2023 after a peak in 2022, on its own more than the entire all-cause incidence gain of 67,627.

Of the 22 level 2 causes, 17 changed their incidence rate. Four rose besides respiratory infections and tuberculosis, namely, diabetes and kidney diseases by 20.9% (15.7 to 26.7), skin and subcutaneous diseases by 9.5% (8.0 to 11.2), sense organ diseases by 8.4% (3.6 to 14.6) and neoplasms by 1.8% (0.7 to 3.4). Twelve fell, headed by nutritional deficiencies with a decrease of 79.1% (52.2 to 90.9), transport injuries with 43.3% (40.4 to 46.2) and cardiovascular diseases with 40.9% (37.9 to 43.4) (Table S12).

Prevalence in 2023 was led by other non-communicable diseases at 71,559 per 100,000 (68,113 to 75,286), then neurological disorders, unintentional injuries, digestive diseases, sense organ diseases and musculoskeletal disorders. Of the 22 level 2 causes, 15 changed their prevalence rate, 4 upward and 11 downward. The four that rose were skin and subcutaneous diseases by 11.8% (10.1 to 13.6), mental disorders by 15.0% (1.3 to 35.4), diabetes and kidney diseases by 12.5% (8.3 to 16.2) and neoplasms by 7.5% (3.4 to 12.9). The largest falls were nutritional deficiencies by 47.5% (18.1 to 65.3), respiratory infections and tuberculosis by 45.4% (28.7 to 54.6) and transport injuries by 43.4% (42.0 to 44.9). Oral disorders led the level 3 prevalence ranking in both years, at 64,392 per 100,000 (59,733 to 69,574) and 57,935 (53,072 to 63,530), followed by headache disorders (Table S13). Table 2 gives the percentage change in prevalence and in incidence for every level 2 cause.

### 3.5. Age and Sex Distribution of Nonfatal Burden

**Figure 3:**
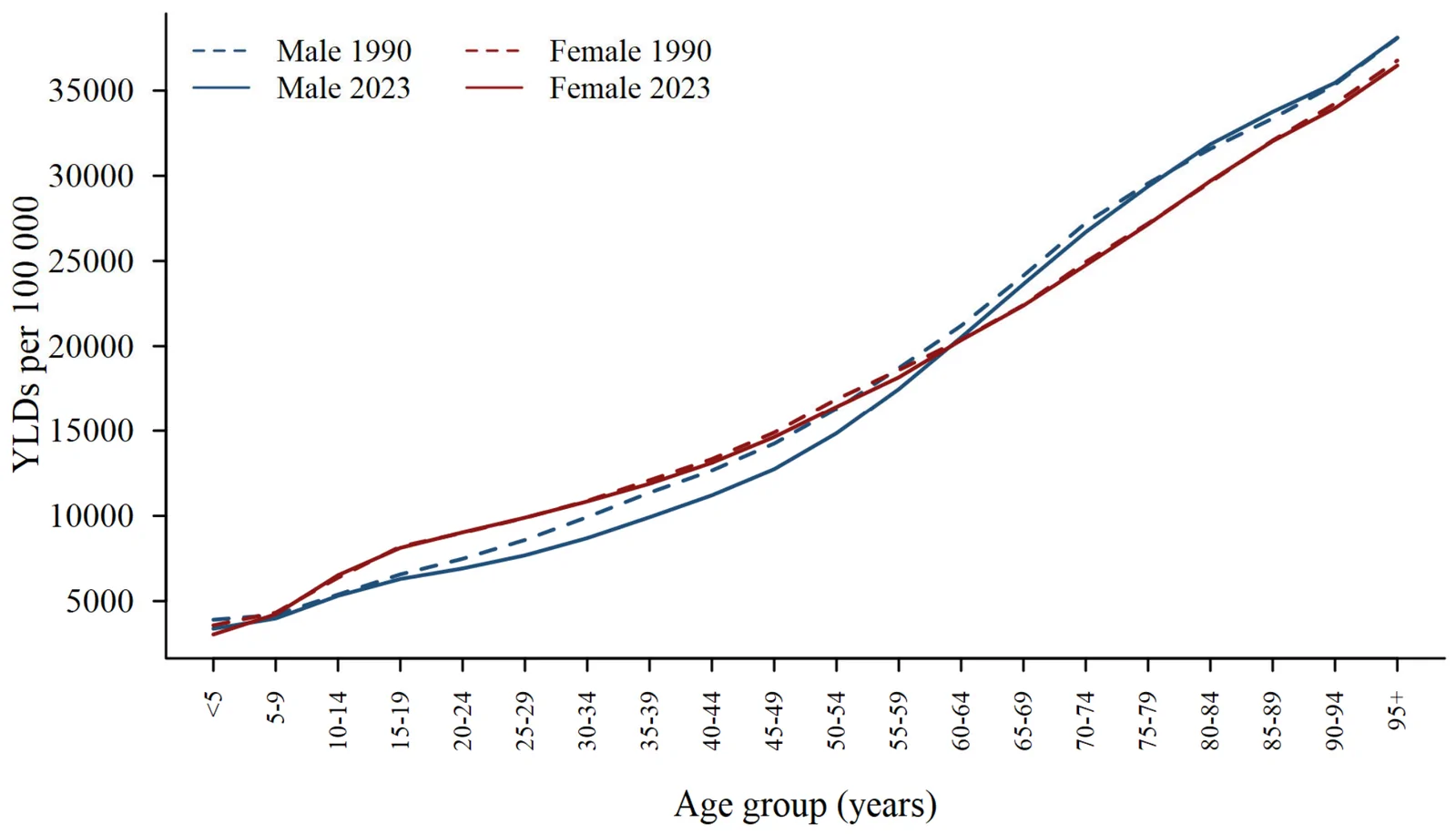
Age-specific rate of years lived with disability from all causes by sex, Romania, 1990 and 2023.

The all-cause rate of years lived with disability rose monotonically across the twenty five-year age groups in both years, from 3213.8 per 100,000 below 5 years to 36,935.6 at 95 years and over in 2023. The ratio between the oldest and the youngest group widened from 9.9 to 11.5 (Figure 3, Table S14).

On point estimates, the age-specific rate fell in seventeen of the twenty groups and rose in three. The largest fall was 14.1%, among children under 5 years, and between 25 and 55 years the falls ran from 5.0% to 7.3%. From 60 years upward, the two years are almost identical, every group lying within 2% of its 1990 rate. The three groups with a higher rate in 2023, those aged 10 to 14, 80 to 84 and 85 to 89 years, exceeded their 1990 values by 0.5%, 0.1% and less than 0.05%, respectively. The share carried by people aged 65 years and over rose from 22.3% to 37.3%, while the share carried by those under 20 years fell from 14.3% to 8.0%.

**Figure 4:**
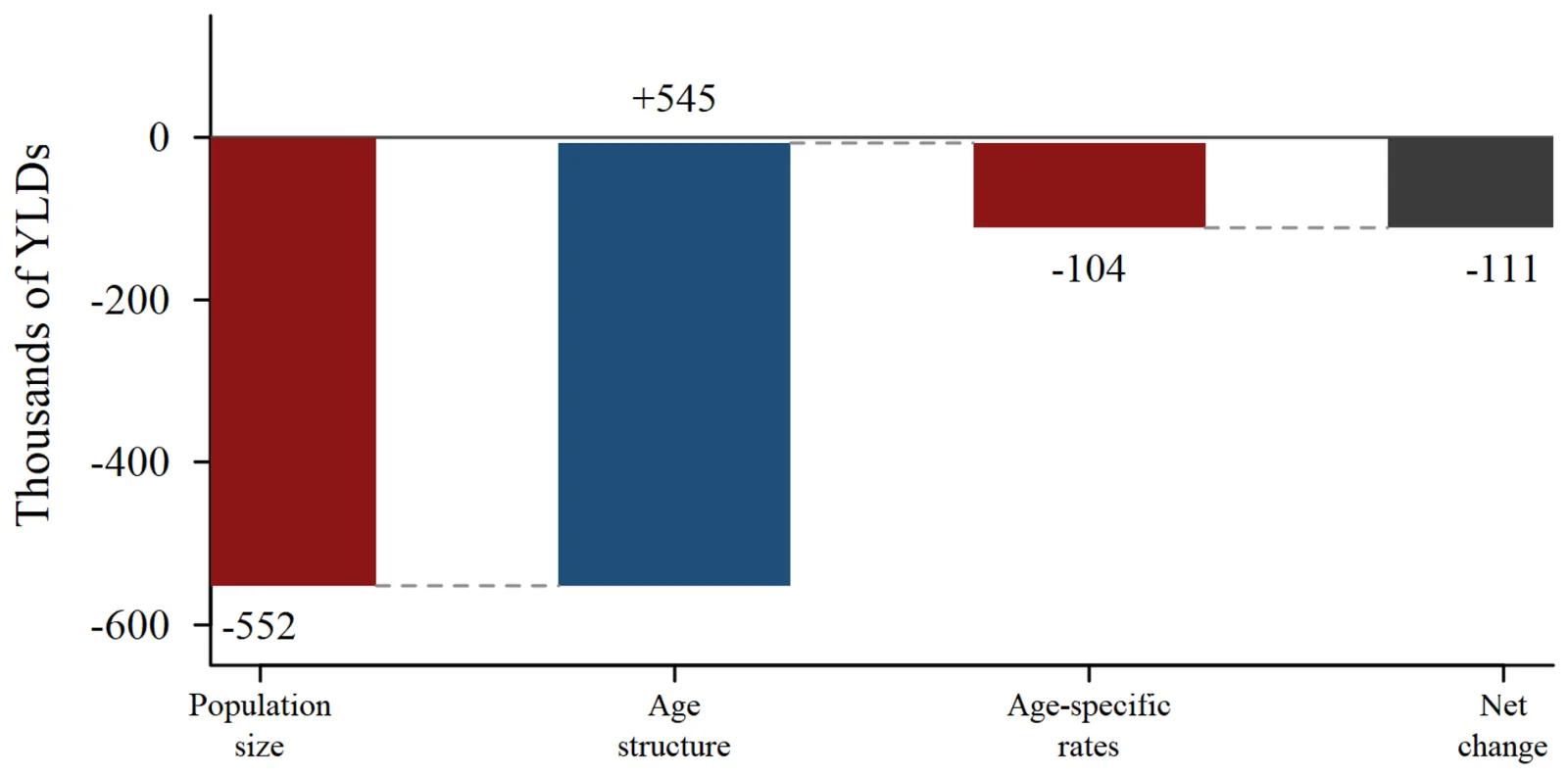
Decomposition of the change in the number of years lived with disability between 1990 and 2023 into population size, age structure and age-specific rates, Romania, both sexes. Red bars reduce the total, the blue bar raises it and the grey bar gives the net change; dashed lines carry the running total between bars. Components are point estimates, in thousands of years lived with disability. No uncertainty intervals were calculated for the components or their balance.

Decomposition yielded approximately −552,000 YLDs for population size, +545,000 for age structure and −104,000 for age-specific rates, summing to a net change of approximately −111,000 YLDs (Figure 4). Population-size and age-structure components were similar in magnitude and opposite in direction, but no uncertainty intervals were calculated for these components or their balance. The 95% UI for the total-count change was −9.2% to +0.7%, leaving its direction uncertain. Net changes by sex were approximately −108,000 YLDs in men and −3000 in women (Table S2).

Age-specific rates were higher in women than in men from 5 to 59 years in 2023, by 30.7% at 20 to 24 years and 10.6% at 50 to 54 years. From 60 years upward, the ordering reversed in every group, the male rate exceeding the female one by 7.2% at 80 to 84 years and 4.5% at 95 years and over. Men also carried the higher rate below 5 years.

### 3.6. Healthy Life Expectancy

HALE at birth in Romania rose by 8.5% (95% UI 7.7 to 9.4), from 60.9 years (58.3 to 63.1) in 1990 to 66.0 (63.1 to 68.4) in 2023, while life expectancy at birth rose from 70.1 years (70.0 to 70.2) to 76.3 (76.1 to 76.4). The two measures gained 5.2 and 6.2 years, respectively, so the point estimate difference between them, denoted here as years lived in poor health, moved from 9.2 to 10.2, an increase of 11.0%. The source does not provide the draw-wise covariance needed to calculate an interval for this difference; the apparent widening is therefore descriptive.

**Figure 5:**
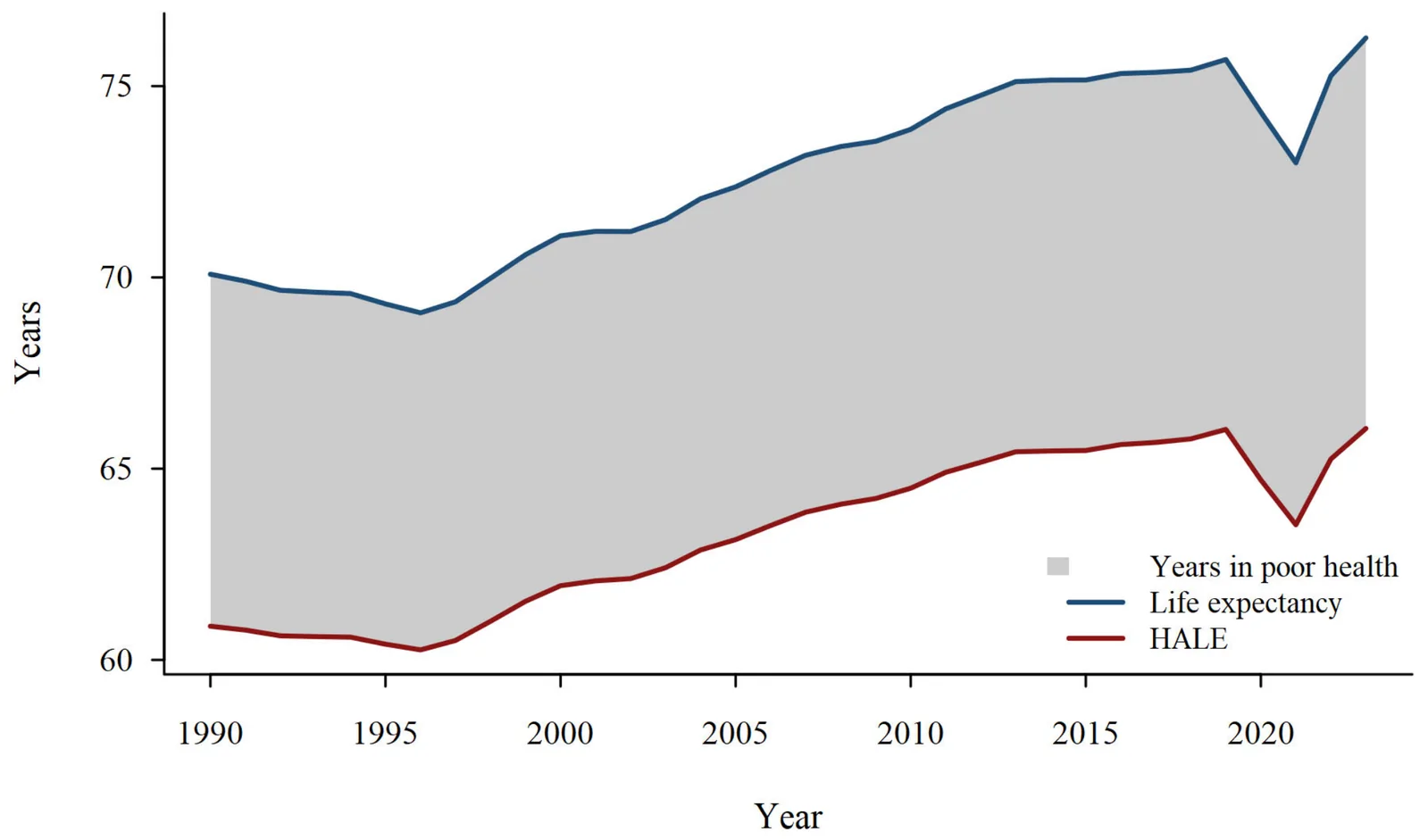
Life expectancy and healthy life expectancy at birth, Romania, both sexes, 1990 to 2023. The shaded area is the difference between the life-expectancy and HALE point estimates, denoted as years lived in poor health. It is not an uncertainty band.

HALE reached its series minimum in 1996, at 60.3 years, and its maximum in 2023 (Figure 5). It gave back 2.5 years between 2019 and 2021, in the pandemic years, and recovered them by 2023. The gap fluctuated, with its largest single-year increase of 0.6 years between 2021 and 2022 (Table S15).

HALE at birth rose by 8.5% (7.8 to 9.5) in men and 8.4% (7.4 to 9.3) in women, which is 5.0 and 5.3 years, while life expectancy gained 5.5 and 6.8 years. On point estimates, years lived in poor health widened by 0.6 years in men and 1.5 in women, reaching 9.0 and 11.5 in 2023, a female excess of 28.6%. At 65 to 69 years, remaining HALE rose by 19.4% (18.0 to 20.6), from 10.5 years (9.5 to 11.4) to 12.6 (11.4 to 13.6), with years in poor health rising from 3.8 to 4.7 (Table S16). Women carried 53.1% of years lived with disability in 1990 and 55.1% in 2023. Their point-estimate count stood nearly unchanged at 1,504,377, while the male count fell by 8.1%; the female age-standardised rate had a change interval that included zero, and the female point-estimate years in poor health widened by 1.5 years against 0.6 in men (Tables S2 and S16). The derived gaps and their sex differences have no uncertainty intervals.

Romania gained 8.5% in HALE between 1990 and 2023 (95% UI 7.7 to 9.4), close to Central Europe, at 8.8% (8.0 to 9.5). Its 2023 point estimate ranked twelfth among the eighteen locations and tenth among the thirteen Central European countries. Every comparator interval overlapped the Romanian interval; no intervals were available for the ranks or between-location differences (Table 3).

The point-estimate difference between life expectancy and HALE in Romania was 10.2 years in 2023, compared with 10.9 in Central Europe and 12.2 in Western Europe. These derived differences have no uncertainty intervals.

### 3.7. Risk-Attributable Nonfatal Burden

The joint distribution of all risk factors accounted for 31.2% (26.5 to 35.4) of years lived with disability in 1990 and 28.3% (24.2 to 32.5) in 2023. The attributable fraction fell by 9.4% of its own value (0.7 to 16.8), which is 2.9 percentage points, and the attributable count fell by 7.0%, from 900,593 years to 837,742. The annual series held above 29% until 2019, dropped to a minimum of 27.5% in 2021 and recovered 0.7 points by 2023 (Table S17).

GBD results returned estimates for 61 of the 65 risks at levels 1 to 3, the four absent being non-optimal temperature with its two components and ambient ozone pollution. The three level 1 groups held their ranks throughout. Of the three, only environmental and occupational risks had a change interval that excluded zero, falling by 27.4% (17.8 to 39.7) to reach 6.3%; behavioural risks stood 13.3% lower and metabolic risks 11.4% higher, both with intervals that include zero. The point estimates nonetheless move metabolic risks from 51% of the behavioural figure in 1990 to 66% of it in 2023.

**Figure 6:**
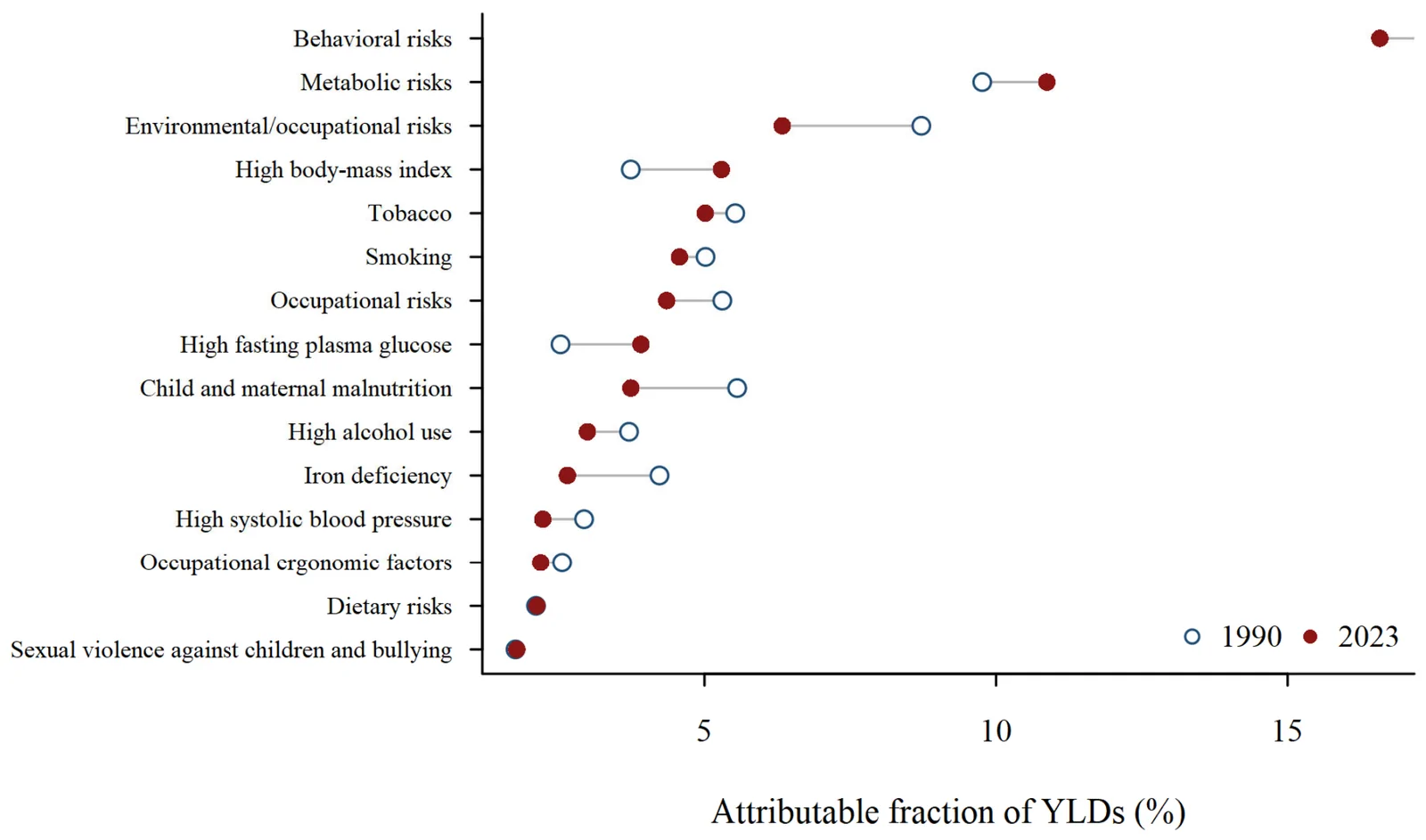
Fifteen largest attributable fractions of years lived with disability by risk factor, Romania, 1990 and 2023, ordered by the 2023 value.

Among the nineteen level 2 risks, high body-mass index had the highest 2023 attributable fraction, 5.3% (1.9 to 8.5), up from fourth place in 1990, and it rose by 41.6% (2.0 to 91.4). Tobacco held second place in both years, at 5.0% in 2023, occupational risks held third at 4.3%, and high fasting plasma glucose climbed from eighth to fourth, at 3.9%, with a rise of 54.5% (25.9 to 83.9). Child and maternal malnutrition, the leading level 2 risk of 1990, fell to fifth, with a point estimate about a third lower and an interval that includes zero. Among the level 3 risks, smoking led in both years, at 4.6% in 2023, followed by iron deficiency at 2.7% and occupational ergonomic factors at 2.2%. Figure 6 shows the fifteen largest attributable fractions; Table S18 gives all 61 risks with their intervals and ranks computed within the hierarchy level.

Thirty-five of the 61 risks changed their attributable fraction over the period, eight upward and 27 downward. Besides high body-mass index and high fasting plasma glucose, the risks that rose were drug use, unsafe sex, kidney dysfunction, a diet high in processed meat, a diet high in sugar-sweetened beverages, and occupational carcinogens, none of them above 1.2% in 2023. Among the risks that fell, the largest in 2023 were high alcohol use, by 19.3% (8.8 to 29.7), and high systolic blood pressure, by 24.1% (4.4 to 40.8); the rest lie below 1.6% and include air pollution, lead exposure and water and sanitation risks. The lower bounds for the high body-mass index increase (2.0%) and the reduction in the joint attributable fraction (0.7%) were close to zero. Table S19 gives the percentage change for every risk.

The point-estimate joint attributable fraction reached 31.9% in men and 25.3% in women in 2023, a male excess of 6.5 percentage points; no draw-wise uncertainty interval for this sex contrast was available. The male value exceeded the female one by 4.7 points for behavioural risks, 3.1 for metabolic risks and 2.2 for environmental and occupational risks (Table 4).

### 3.8. Risk Profile Against Comparator Locations

The joint fraction of years lived with disability attributable to all risks stood at 28.3% in Romania in 2023, within a range running from 26.4% in Western Europe to 30.7% in Poland across the eighteen locations. Ordered from the highest fraction downward, Romania placed fourteenth of the eighteen and eleventh of the thirteen Central European countries, 1.2 percentage points under the Central Europe estimate, 0.2 above Eastern Europe and 2.0 under the global one. The Romanian value was 31.2% in 1990 and the same ordering placed it ninth, 0.8 points under Central Europe. The eighteen locations span 4.3 percentage points in 2023 and 4.6 in 1990, so the positions separate them less than the rank numbers suggest (Table S20). Six of the eighteen changed over the period, Romania being among them, with a fall of 9.4% seen in its own value (0.7 to 16.8); Western Europe changed by 8.6% (1.8 to 15.5) and the global aggregate changed by 6.4% (0.7 to 11.7), while the Central European and Eastern European change intervals included zero. No uncertainty intervals were available for ranks or between-location differences.

The comparison with Central Europe covers the 22 risks reported at levels 1 and 2. Romania carried the higher attributable fraction for eight of them and the lower one for the remaining fourteen. The three widest gaps fell on child and maternal malnutrition, at 3.74% of years lived with disability in Romania against 3.17% for the region, on occupational risks, at 4.35% against 4.00%, and on kidney dysfunction, at 1.13% against 0.86%. Kidney dysfunction carries the smallest of the three fractions and the largest proportional gap, the Romanian value standing at 1.31 times the regional one.

**Figure 7:**
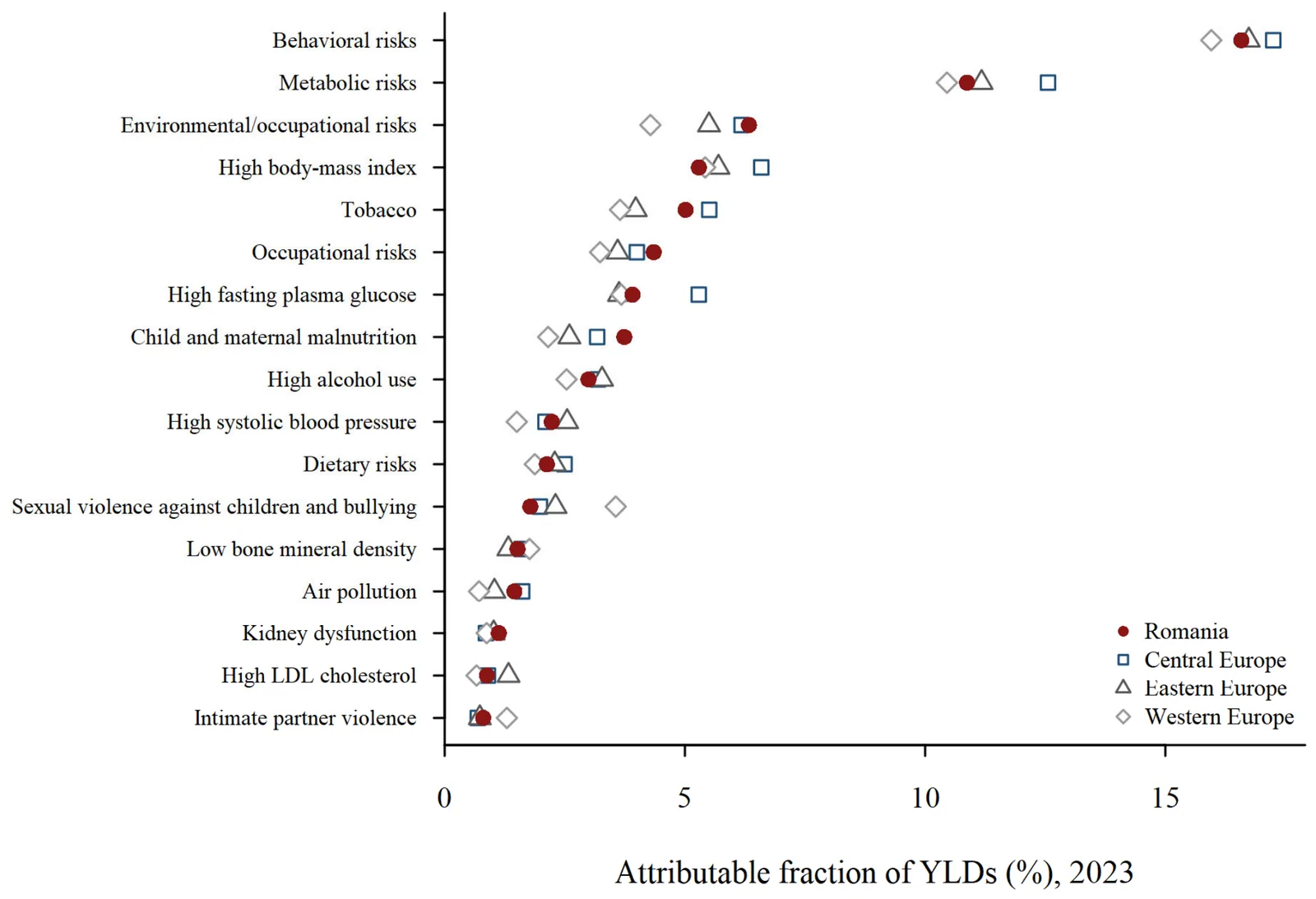
Attributable fraction of years lived with disability by risk factor in 2023, Romania against the Central Europe, Eastern Europe and Western Europe regional estimates. Risks with a Romanian value below 0.5% are omitted; the rule excludes drug use, at 0.49% in Romania against 1.46% in Western Europe, one of the two risks on which the Romanian fraction sits furthest below the Western European one. The full set appears in Table S21.

Among the fourteen risks for which the Romanian fraction was the lower one, the widest gap fell on the metabolic group, at 10.9% of years lived with disability against 12.6% for Central Europe. The same direction holds for its two largest components, high fasting plasma glucose at 3.9% against 5.3%, and high body-mass index at 5.3% against 6.6%. Behavioural risks stood at 16.6% against 17.2%, tobacco at 5.0% against 5.5% and dietary risks at 2.1% against 2.5% (Figure 7, Table S21).

Eight risks separated Romania from Western Europe by more than 0.7 percentage points in 2023, six with the higher fraction in Romania and two with the higher fraction in Western Europe. The environmental and occupational group stood at 6.3% of years lived with disability against 4.3%, child and maternal malnutrition at 3.7% against 2.1%, tobacco at 5.0% against 3.6%, occupational risks at 4.3% against 3.2%, air pollution at 1.4% against 0.7% and high systolic blood pressure at 2.2% against 1.5%. In the other direction, sexual violence against children and bullying stood at 1.8% in Romania against 3.6% in Western Europe, the second widest gap among the risks in Table S21, and drug use at 0.5% against 1.5%. Air pollution carries the smallest of the six Romanian excesses and the widest proportional gap, the Romanian value standing at 2.0 times the Western European one.

The summary exposure value for all risks combined stood at 24.0% for Romania in 2023, seventh of the eighteen locations when they are ordered from the highest exposure downward. Czechia headed the set at 26.8% and Slovakia closed it at 22.1%, placing the Romanian value 10.2% under the first and 8.8% over the last. It stood 1.6% over the Central Europe estimate, 3.3% over Eastern Europe and 3.7% under Western Europe, which held the third highest value of the eighteen (Table S22). The Romanian change was 3.8% (95% UI −3.9 to 11.9). Its interval and those of the three regional aggregates included zero, while the global estimate fell by 3.8% (0.5 to 7.4).

## 4. Discussion

Romania’s crude YLD rate rose by 17.0% between 1990 and 2023. The age-standardised rate was 4.1% lower, although its 95% UI for change, −10.1% to +1.3%, included zero. Across the cause and risk comparisons, 144 of 257 change intervals excluded zero. Some increases had lower bounds close to zero, including mental disorders (2.4%), anxiety disorders (4.5%) and high body-mass index attribution (2.0%). The joint fraction attributed to evaluated risks fell from 31.2% to 28.3%.

Globally, age-standardised YLD rates rose by 2.3% between 2010 and 2023, while age-standardised YLL rates fell by 18.8% [1]. Across the EU-27, age-standardised YLD rates remained largely unchanged between 2010 and 2019 as death and YLL rates declined [7]. In Poland, age-standardised YLD rates fell by 4.0% between 1990 and 2017, compared with a 46.0% decline in YLL rates [20]. Among Europeans aged 65 years and older, YLL trends were driven mainly by cardiovascular disease, whereas YLD rates showed little decline over the past three decades [21]. Romania’s estimated YLD rate change was also small, but this study did not analyse national mortality trends.

Musculoskeletal disorders remained the leading level 2 cause of years lived with disability in both years, and low-back pain remained the leading level 3 cause in both years and both sexes, consistent with the wider evidence that low-back pain remains the main cause of YLDs globally [22]. GBD 2021 estimated the global age-standardised YLD rate for low-back pain at 832 per 100,000 in 2020, and this rate fell by 10.5% between 1990 and 2020 [22]. The Romanian rate of 1432.1 per 100,000 in 2023 and the 6.2% decline reported here were estimated in GBD 2023. Azhati et al. analysed low-back pain in Chinese adults aged 45 years and older using GBD 2023 estimates. Absolute YLDs increased despite declining age-restricted age-standardised rates; high body-mass index was the only evaluated risk with an increasing age-standardised attributable YLD rate [23]. Their decomposition separates growth of the population aged 45 years and older, ageing within that population and rate changes. Their age-restricted rates cannot be compared directly with the all-age Romanian rates. Modifiable risk factors explained 38.8% of global low-back pain YLDs, with the attributable burden specifically linked to occupational ergonomic factors, smoking, and high BMI [22]. Romania’s occupational risk PAF was 1.1 percentage points above the Western European estimate; no interval was available for this difference.

On point estimates, anxiety disorders moved from fifth to second among level 3 causes, and mental disorders overtook unintentional injuries to rank second among level 2 causes. The lower bounds for their rate increases were 4.5% and 2.4%, respectively; the ranks have no uncertainty intervals. In global estimates, anxiety disorders were already among the leading contributors to mental health burden before the pandemic, remained one of the highest-burden mental disorder subtypes, and showed the largest increase in age-standardised burden rates among major mental disorder categories [1,24,25]. Longer-run GBD 2021 analyses show overall increases in age-standardised incidence, prevalence, and YLDs or DALYs from the 1990s to 2021, with a particularly sharp rise from 2019 to 2021 [26,27]. Two pandemic analyses place part of that increase in 2020 and 2021: a global meta-regression estimated 76.2 million additional anxiety disorder cases in 2020, a 25.6% increase over the pre-pandemic baseline, with larger increases in females and younger age groups and in locations with greater mobility restriction and higher SARS-CoV-2 infection rates [28]. A counterfactual GBD 2021 time-series analysis likewise estimated that the observed age-standardised DALY rate from anxiety disorders was 14.3% above the rate expected without the pandemic in 2020–2021, one of the most prominent excess increases among 174 causes, with the burden increase again more pronounced in females, being the greatest in absolute terms at ages 15–49 years, and largest in relative terms at ages 5–14 years [29]. The Romanian female rate being 89.9% higher than the male rate in 2023 is consistent with the wider literature showing that anxiety disorder burden is persistently higher in women than in men: GBD 2019 estimated female prevalence and DALY rates at about 1.6 times those of males, GBD 2021 analyses found a continued female excess and a widening sex gap in incidence, and broader reviews also report higher female prevalence and greater burden of illness [30,31,32,33].

Neoplasms rose by 43.0% (20.0 to 72.8) on the age-standardised YLD rate while their incidence rose by 1.8% (0.7 to 3.4). A higher YLD rate can reflect incidence, survival, duration with disability, ascertainment or disability severity. These aggregate estimates cannot separate those mechanisms; therefore, the divergence should not be interpreted as evidence of either improved or worsened oncological outcomes [5].

Romania showed increasing YLD attribution to high body-mass index and high fasting plasma glucose, although their 2023 PAF point estimates remained below those of Central Europe. In Romania, diabetes mellitus increased by 55.0% (43.4 to 67.8) during the selected timespan, and rose from 18th to 9th among level 3 causes; over the same period, high fasting plasma glucose increased by 54.5% (25.9 to 83.9) and moved from eighth to fourth among level 2 risk factors on point estimates, while high body-mass index increased by 41.6% (2.0 to 91.4) and became the leading level 2 risk factor on point estimates. Globally, metabolic risks were the only level 1 risk category for which attributable DALY counts increased between 2010 and 2023, rising by 30.7% (24.8 to 37.3), even as the age-standardised DALY rate attributable to metabolic risks fell by 6.7% (2.0 to 11.0) [1]. Among the 25 leading level 3 risks worldwide, the age-standardised attributable DALY rate increased clearly for high BMI by 10.5% (0.1 to 20.9) and for drug use by 8.4% (2.6 to 15.3), whereas the increase for high fasting plasma glucose remained directionally uncertain [1]. Anthropometric surveys found that adult obesity prevalence increased from 1990 to 2022 in 188 countries for women and all but one country for men, with some of the largest increases in central Europe, including Romania, among men [34].

Romania’s population fell by 17.9% between 1990 and 2023, while the crude YLD rate rose by 17.0%. The total YLD estimate consequently changed little. GBD analyses report counts alongside age-standardised rates because population changes can produce different trends in these measures [1,6]. Decomposition studies quantify the contributions of population size, age structure and age-specific rates [35,36,37]. Here, the population size and age structure components were approximately −552,000 and +545,000 YLDs. Their apparent balance is a point estimate result without component uncertainty intervals, and the interval for the total count change includes zero. Analyses of older European populations also show that absolute burden depends on population size and age structure [21]. Forecasts of care requirements need to account for the increase in the share of YLDs at ages 65 years and older, from 22.3% to 37.3% [6].

GBD analyses estimate disease burden by age, sex and location [1,21,38]. In Romania, the fractions attributed to high body-mass index and high fasting plasma glucose increased between 1990 and 2023. PREDATORR estimated chronic kidney disease in 6.7% of adults and found associations with impaired glucose regulation, hyperuricaemia and hypertriglyceridaemia [13]. The Romanian Health Evaluation Survey found higher body-mass index, blood pressure and fasting plasma glucose in rural than urban residents [14]. The national GBD estimates do not distinguish rural from urban populations.

Romania’s HALE gain of 8.5% between 1990 and 2023 was close to Central Europe’s 8.8% and above Western Europe’s 5.4% [1]. Convergence would mean a narrowing difference in HALE between locations over time. The percentage gains alone do not show whether that difference narrowed. In the GBD estimates used here, Romanian life expectancy rose by 6.2 years and HALE by 5.2 years [1]. Their difference increased from 9.2 to 10.2 years, but the absence of matched draws prevented the estimation of an interval for this change.

The GBD estimates should be compared with Romanian disease registries and repeated national surveys using the same age groups, years and case definitions. Such comparisons could identify conditions for which national data differ most from the modelled estimates. Regional and rural–urban analyses could then examine differences in disease burden and access to diagnosis and treatment. Studies of specific policy changes would need measurements before and after implementation and an appropriate comparison population. Matched GBD draws would allow intervals to be calculated for the decomposition, the life expectancy–HALE gap and between-location differences.

## 5. Limitations

Every figure reported here is a modelled estimate reused from a published round, so the analysis inherits whatever the estimation carries. The uncertainty intervals propagate sampling and parameter uncertainty through 250 draws at every step, and they do not cover the risk of bias in the input data, the choice of model form or the assumptions behind the cause and risk hierarchies. Each round re-estimates the whole series from 1990, so the values used here belong to GBD 2023 and do not align term by term with values published in earlier rounds, which sets the limit on any comparison with the GBD 2021 estimate for low-back pain.

Prevalence and incidence for Romania rest on thin national input and are fitted with a Bayesian meta-regression that borrows strength across the region when country data are sparse, so a Romanian estimate can sit closer to the Central European fit than a national survey would place it. Therefore, the distances reported between Romania and its region, which are of the order of one to two percentage points on the attributable fractions, are partly a property of the estimation, on top of any real difference between populations. Larger distances from Western Europe and the global aggregate do not remove this dependence on model structure. National registries and routine coding and reporting remain incomplete for several conditions, which may further limit national input coverage.

Conditions whose prevalence is measured through contact with health services carry a further problem over a window of 33 years, namely, access to care and diagnostic practice both changed, so rising ascertainment can lift a modelled prevalence with no change in the underlying condition. The increases reported for anxiety disorders and diabetes are read with this in mind, because both causes draw heavily on contact-based sources.

The results tool returned attributable nonfatal burden for 61 of the 65 risks at levels 1 to 3, with non-optimal temperature, its two components and ambient ozone pollution carrying an exposure estimate and no attributable years lived with disability, so coverage is incomplete; neither the direction nor the magnitude of any effect on the aggregate can be determined from these data. Attributable fractions cannot be added across risks because their effects overlap and some operate through intermediate risks; the GBD joint estimates account for mediation rather than simply summing the separate fractions. The cross-check against the capstone appendix returned wider intervals for the aggregate risk levels than the results tool serves, so the intervals given here for level 1 and level 2 risks are the narrower of the two and any comparison at those levels should be read with that in mind.

The capstone sets national estimates against the values expected from the Socio-demographic Index, which separates what a population attains from what its level of development predicts; that comparison is absent here because the results tool does not serve expected values, so the analysis across locations reports observed values only. A distance between Romania and Czechia or Slovenia therefore mixes development with health system performance, and no part of the distances reported here can be assigned to either. The tool does not compute differences between locations either, so those distances are reported between point estimates with a statement of interval overlap, and the location rankings describe positions in a single year. In the same way, an interval for percentage change that excludes zero indicates an increase or decrease, and two changes whose intervals overlap are not thereby shown to be equal.

Disability weights come from population surveys carried out mostly outside Romania, and applying them here assumes that valuations of health states transport across settings. GBD adjusts cause-specific years lived with disability for comorbidity. Cause categories at different hierarchy levels overlap and should not be summed across levels; the reported shares use the all-cause total as their denominator. The decomposition is arithmetic and carries no causal content; the population, age structure and rate components are not independent processes, and the symmetric split used is one of several defensible ones. Matched draws were unavailable for estimating uncertainty in the decomposition. Migration, fertility and survival affect population size and age structure, but their separate contributions were not estimated.

Rural and urban populations, the eight development regions and the socioeconomic strata that measured surveys separately are invisible in the single national value that GBD 2023 produces for Romania, and the national estimate averages over differences in exposure that direct measurement finds to be substantial.

## 6. Conclusions

Romania’s crude YLD rate increased from 120.7 to 141.2 per 1000 population between 1990 and 2023, a rise of 17.0%. The share of YLDs at ages 65 years and older rose from 22.3% to 37.3%, while the share before age 20 fell from 14.3% to 8.0%. Musculoskeletal disorders remained the leading cause group, anxiety disorders and diabetes increased, and injuries declined. The joint risk-attributable fraction fell from 31.2% to 28.3%. Population contraction and ageing made opposing contributions of similar sizes in the point-estimate decomposition; uncertainty in their balance was not assessed. The intervals for changes in the age-standardised rate and the total YLD count both included zero.

The greater share of disability at older ages has implications for the rehabilitation, long-term care and management of musculoskeletal disease. The increases in anxiety disorders and diabetes also warrant attention in mental-health and metabolic care. Changes in exposures, diagnostic practice, healthcare access and public health policy are possible contributors to the trends observed over these 33 years; their effects require national and regional studies with data on those factors. Mortality trends should be examined alongside nonfatal burden in those studies.

## Figures and Tables

**Table 1:** Twenty largest level 3 causes of years lived with disability, age-standardised rate per 100,000 population, Romania, 2023.

Cause	Rank 2023	Rate 2023 (95% UI)	Rank 1990	Rate 1990 (95% UI)
Low-back pain	1	1432.1 (1016.9 to 1930.3)	1	1526.3 (1095.6 to 2054.6)
Anxiety disorders	2	688.8 (425.3 to 1033.6)	5	486.1 (309.7 to 720.4)
Falls	3	610.1 (436.8 to 832.2)	2	695.8 (508.8 to 936.9)
Age-related and other hearing loss	4	551.0 (386.9 to 740.3)	3	555.6 (391.5 to 744.1)
Headache disorders	5	536.1 (369.5 to 733.1)	4	537.3 (366.5 to 731.5)
Neonatal disorders	6	356.5 (257.2 to 466.3)	9	324.1 (226.7 to 442.3)
Depressive disorders	7	340.9 (235.6 to 477.8)	8	324.1 (220.6 to 451.4)
Exposure to mechanical forces	8	338.7 (221.7 to 498.7)	7	463.0 (327.7 to 664.1)
Diabetes mellitus	9	306.5 (211.5 to 424.8)	18	197.8 (134.0 to 270.4)
Oral disorders	10	293.9 (181.6 to 435.1)	10	320.8 (201.1 to 474.2)
Dietary iron deficiency	11	286.1 (147.6 to 560.9)	6	477.9 (214.8 to 982.7)
Gynaecological diseases	12	235.2 (157.0 to 329.9)	13	243.1 (160.4 to 338.9)
Neck pain	13	229.9 (154.8 to 336.0)	15	231.8 (153.7 to 342.0)
Osteoarthritis	14	228.9 (107.7 to 498.5)	16	207.0 (97.1 to 456.1)
Chronic obstructive pulmonary disease	15	212.8 (171.4 to 252.2)	14	231.9 (182.9 to 278.7)
Alcohol use disorders	16	197.4 (136.1 to 279.9)	11	258.9 (179.5 to 360.3)
COVID-19	17	193.5 (61.5 to 477.8)	Not applicable	0.0 (0.0 to 0.0)
Schizophrenia	18	187.1 (131.3 to 244.7)	19	185.9 (130.8 to 238.7)
Stroke	19	173.6 (124.5 to 222.1)	12	255.5 (179.2 to 325.9)
Asthma	20	163.4 (100.6 to 238.2)	23	158.9 (97.0 to 230.5)

**Table 2:** Percentage changes in age-standardised prevalence and incidence by level 2 cause, Romania, 1990 to 2023.

Cause	Prevalence Change, % (95% UI)	Incidence Change, % (95% UI)
All causes	−2.5 (−3.2 to −1.8)	+19.2 (−2.7 to 64.1)
Cardiovascular diseases	−17.5 (−20.8 to −13.9)	−40.9 (−43.4 to −37.9)
Chronic respiratory diseases	−1.5 (−6.9 to 3.3)	−1.4 (−6.2 to 2.6)
Diabetes and kidney diseases	+12.5 (8.3 to 16.2)	+20.9 (15.7 to 26.7)
Digestive diseases	−8.0 (−9.6 to −6.4)	−4.7 (−6.6 to −2.9)
Enteric infections	−36.0 (−39.7 to −31.8)	−25.4 (−29.8 to −20.2)
HIV/AIDS and sexually transmitted infections	+0.9 (−2.9 to 6.1)	−1.4 (−8.1 to 6.1)
Maternal and neonatal disorders	+16.9 (−6.0 to 43.4)	−14.4 (−20.2 to −7.4)
Mental disorders	+15.0 (1.3 to 35.4)	−9.7 (−21.8 to 4.6)
Musculoskeletal disorders	+0.5 (−1.9 to 2.7)	−3.4 (−6.0 to −0.6)
Neglected tropical diseases and malaria	−32.6 (−54.5 to 6.5)	−31.4 (−51.2 to −15.3)
Neoplasms	+7.5 (3.4 to 12.9)	+1.8 (0.7 to 3.4)
Neurological disorders	−0.1 (−0.7 to 0.6)	−0.0 (−0.5 to 0.5)
Nutritional deficiencies	−47.5 (−65.3 to −18.1)	−79.1 (−90.9 to −52.2)
Other infectious diseases	−33.2 (−52.5 to −0.3)	−16.4 (−20.9 to −11.4)
Other non-communicable diseases	−6.1 (−8.4 to −3.4)	−8.5 (−17.4 to 3.5)
Respiratory infections and tuberculosis	−45.4 (−54.6 to −28.7)	+64.1 (9.5 to 176.4)
Self-harm and interpersonal violence	−8.5 (−11.6 to −5.3)	−18.5 (−25.0 to −10.7)
Sense organ diseases	+0.0 (−1.5 to 1.7)	+8.4 (3.6 to 14.6)
Skin and subcutaneous diseases	+11.8 (10.1 to 13.6)	+9.5 (8.0 to 11.2)
Substance use disorders	−21.0 (−31.3 to −10.3)	−18.3 (−28.8 to −7.2)
Transport injuries	−43.4 (−44.9 to −42.0)	−43.3 (−46.2 to −40.4)
Unintentional injuries	−13.8 (−15.3 to −12.4)	−17.2 (−20.8 to −13.4)

UI, uncertainty interval. Changes are relative to the 1990 rates. An interval containing zero indicates uncertainty about the direction of change.

**Table 3:** Healthy life expectancy and life expectancy at birth, both sexes, 2023, eighteen locations. Locations are ordered by the HALE point estimate. Ranks and years in poor health have no uncertainty intervals.

Location	HALE 2023 (95% UI)	Life Expectancy 2023 (95% UI)	Years in Poor Health
Western Europe	69.82 (66.49 to 72.66)	82.03 (81.98 to 82.08)	12.21
Slovenia	69.32 (66.02 to 72.19)	81.64 (81.45 to 81.84)	12.32
Albania	68.57 (65.39 to 71.28)	79.64 (79.19 to 80.10)	11.07
Czechia	68.09 (64.85 to 70.80)	79.88 (79.76 to 79.99)	11.78
Poland	67.31 (64.12 to 70.00)	78.64 (78.55 to 78.73)	11.33
Croatia	67.28 (64.18 to 69.90)	78.58 (78.37 to 78.79)	11.30
Slovakia	67.21 (64.15 to 69.78)	78.15 (77.99 to 78.32)	10.95
Central Europe	66.83 (63.76 to 69.42)	77.74 (77.67 to 77.82)	10.91
Bosnia and Herzegovina	66.55 (63.37 to 69.16)	77.71 (77.20 to 78.21)	11.16
North Macedonia	66.44 (63.51 to 68.78)	77.03 (76.65 to 77.39)	10.59
Montenegro	66.32 (63.30 to 68.90)	77.05 (76.80 to 77.34)	10.73
Romania	66.05 (63.15 to 68.41)	76.26 (76.13 to 76.39)	10.21
Hungary	66.05 (63.04 to 68.57)	76.82 (76.69 to 76.96)	10.78
Serbia	65.99 (63.27 to 68.55)	75.98 (75.38 to 76.56)	9.99
Bulgaria	65.64 (62.53 to 68.13)	75.92 (75.73 to 76.09)	10.28
Central Europe, Eastern Europe, and Central Asia	63.97 (61.04 to 66.41)	74.35 (74.11 to 74.54)	10.38
Global	63.14 (60.15 to 65.61)	73.84 (73.62 to 74.06)	10.70
Eastern Europe	62.97 (60.05 to 65.41)	73.32 (72.83 to 73.68)	10.35

**Table 4:** Fractions of years lived with disability attributable to selected risk factors and risk groups by sex, Romania, 2023.

Risk Factor or Group	Male, %	Female, %	Male Minus Female, pp
All risk factors	31.88	25.34	+6.54
Behavioral risks	19.24	14.55	+4.69
Metabolic risks	12.62	9.48	+3.13
Environmental/occupational risks	7.51	5.28	+2.23
High body-mass index	5.73	4.89	+0.84
Tobacco	6.73	3.68	+3.05
Smoking	6.30	3.23	+3.07
Occupational risks	5.16	3.60	+1.55
High fasting plasma glucose	4.96	3.08	+1.88
Child and maternal malnutrition	3.03	4.37	−1.34
High alcohol use	4.53	1.70	+2.83
Iron deficiency	2.02	3.23	−1.21
High systolic blood pressure	2.93	1.71	+1.22
Occupational ergonomic factors	2.17	2.20	−0.03
Dietary risks	3.01	1.44	+1.58
Sexual violence against children and bullying	2.16	1.44	+0.72
Low bone mineral density	1.77	1.32	+0.44
Air pollution	1.69	1.25	+0.43
Particulate matter pollution	1.67	1.24	+0.43
Sexual violence against children	1.66	1.11	+0.55
Occupational injuries	1.86	0.65	+1.21
Kidney dysfunction	1.45	0.88	+0.57
Low birth weight and short gestation	0.98	1.10	−0.12
High LDL cholesterol	1.15	0.66	+0.49
Intimate partner violence	Not applicable	1.53	Not estimable

Values are point estimates. pp, percentage points. Risk groups and individual risks overlap and should not be summed. No uncertainty intervals for sex contrasts were available. The male estimate for intimate partner violence is not applicable.

## Data Availability

All source estimates analysed in this study are publicly available through the GBD Results Tool. The supporting tabulations are included in the Supplementary Materials. The analysis code is available from the corresponding author upon reasonable request.

## Supplementary Materials

The following supporting information can be downloaded at: https://www.mdpi.com/article/10.3390/healthcare14183035/s1.
