# Supplementary material for: Nonfatal Burden of Disease and Risk-Attributable Burden in Romania, 1990 to 2023: A Secondary Analysis of Global Burden of Disease 2023 Estimates
Source: Healthcare (Basel). 2026 Sep 16;14(18):3035. doi: 10.3390/healthcare14183035 (PMC13606279; doi:10.3390/healthcare14183035)
Supplement: Supplementary file 1 [file healthcare-14-03035-s001.zip › healthcare-4538797-supplementary.pdf]

# Supplementary

## *Nonfatal burden of disease and risk-attributable burden in Romania, 1990 to 2023: a secondary analysis of Global Burden of Disease 2023 estimates*

All estimates come from the Global Burden of Disease Study 2023 results tool, extracted on 4 and 5 August 2026. Rates are per 100,000 population unless stated otherwise, and uncertainty intervals are the 95% intervals reported by the tool, *summarising the 250 draws the source propagates through the estimation. Values shown without an interval, among them ranks, sex contrasts, years in poor health and the decomposition components, are point-estimate quantities and carry no uncertainty interval.*

## Contents


Table S1. GATHER checklist and the location of each item in the manuscript.
Table S2. Point-estimate decomposition of the change in years lived with disability between 1990 and 2023, Romania. Component uncertainty intervals were not calculated. Displayed totals imply a change of -111,155 years lived with disability; the rounded components sum to -111,156.
Table S3. Age-standardised rate of years lived with disability from all causes by sex, Romania, 1990 to 2023.
Table S4. COVID-19 age-standardised rates of years lived with disability and of incidence, Romania, 2020 to 2023.
Table S5. Age-standardised rate of years lived with disability from all causes, eighteen locations, 1990 and 2023.
Table S6. Percentage change in the four headline measures, eighteen locations, 1990 to 2023.
Table S7. Age-standardised rate of years lived with disability per 100 000 population by level 2 cause, Romania, 1990 and 2023. This table provides the ranks and uncertainty intervals underlying Figure 2 in the manuscript.
Table S8. Percentage change in the age-standardised rate of years lived with disability by level 2 cause, Romania, 1990 to 2023.
Table S9. Age-standardised rate of years lived with disability by level 3 cause, Romania, 1990 and 2023.
Table S10. Twenty-five largest level 3 causes by sex, age-standardised rate, Romania, 2023.
Table S11. Percentage change in the age-standardised rate of years lived with disability by level 3 cause, Romania, 1990 to 2023.
Table S12. Age-standardised incidence by level 2 cause, Romania, 1990 and 2023.
Table S13. Age-standardised prevalence by level 2 cause, Romania, 1990 and 2023.
Table S14. Age-specific rate of years lived with disability from all causes, Romania, 1990 and 2023.
Table S15. Healthy life expectancy and life expectancy at birth, Romania, both sexes, 1990 to 2023. Years in poor health is the point-estimate difference between life expectancy and HALE; no uncertainty interval for this correlated difference was calculated.
Table S16. Healthy life expectancy and life expectancy at birth and at 65 to 69 years by sex, Romania, 1990 and 2023.
Table S17. Attributable fraction and attributable rate of years lived with disability, all risk factors, Romania, 1990 to 2023.
Table S18. Attributable fraction of years lived with disability by risk factor, Romania, 1990 and 2023.
Table S19. Percentage change in the attributable fraction of years lived with disability by risk factor, Romania, 1990 to 2023. Relative changes become unstable when the 1990 fraction lies near zero and are read together with the absolute percentage-point changes.
Table S20. Attributable fraction of years lived with disability, all risk factors, eighteen locations, 1990 and 2023.
Table S21. Attributable fraction of years lived with disability by risk factor, Romania against regional aggregates, 2023.
Table S22. Summary exposure value for all risk factors, eighteen locations, 1990 and 2023.

**Table S1. GATHER checklist and the location of each item in the manuscript.**

| Item | Checklist item | Where reported |
|---|---|---|
| 1 | Definition of indicators, populations and time periods | Section 2.2 for measures, 2.3 for cause and risk hierarchies, 2.4 for locations, 2.5 for the time window |
| 2 | Rationale for choice of data sources | Section 2.1. The analysis reuses a single source, the GBD 2023 estimates, so no source selection was performed |
| 3 | Sources accessed and dates | Section 2.1. All estimates were extracted from the GBD Results tool on 4 and 5 August 2026 |
| 4 | Identifiable data | No individual or identifiable data were used |
| 5 | Non-identifiable data available to others | All estimates are public through the GBD Results tool and can be retrieved with the specifications given in sections 2.2 to 2.5 |
| 6 | Description of steps taken to correct data | No correction was applied by the authors; the corrections built into the source estimates are described in the capstone |
| 7 | Methods description in sufficient detail | Sections 2.2 and 2.6 for the analysis; the estimation methods are in the capstone and its appendices |
| 8 | Explanation of changes from previous versions | This is the first analysis of nonfatal and risk-attributable burden for Romania in this series |
| 9 | Source code or description of computations | Section 2.6. Extraction, analysis and figure code are archived with the manuscript |
| 10 | Estimates with uncertainty intervals | All estimates are reported with the 95% uncertainty intervals of the source, in the text and in the tables |
| 11 | How uncertainty was propagated | *Section 2.2. GBD 2023 summarises 250 uncertainty draws; the propagation was performed within the source, and the present analysis reuses the published summaries* |
| 12 | Reporting of limitations | Section 5, which covers ascertainment, the aggregation of uncertainty at higher hierarchy levels and the absence of an uncertainty interval for differences |
| 13 | Statement on funding and conflicts | Declarations section |
| 14 | Data sharing statement | Section 2.7 |
| 15 | Analysis by subgroups | Results are reported by sex and by age group throughout, and by location in sections 3.1, 3.6 and 3.8 |
| 16 | Statement of ethical approval | Section 2.7 |
| 17 | Preferred citation of the source estimates | Section 2.1 and the reference list |
| 18 | Reporting of the standard population used | Section 2.2, the GBD world standard population |

**Table S2.** ***Point-estimate decomposition of the change in years lived with disability between 1990 and 2023, Romania. Component uncertainty intervals were not calculated. Displayed totals imply a change of -111,155 years lived with disability; the rounded components sum to -111,156.***

| Population | Total 1990 | Total 2023 | Net change | Population size | Age structure | Age-specific rates |
|---|---|---|---|---|---|---|
| Both sexes | 2,840,268 | 2,729,113 | -111,156 | -551,978 | +545,080 | -104,258 |
| Male | 1,332,609 | 1,224,736 | -107,873 | -270,076 | +247,783 | -85,580 |
| Female | 1,507,659 | 1,504,377 | -3,282 | -279,594 | +292,675 | -16,363 |

**Table S3. Age-standardised rate of years lived with disability from all causes by sex, Romania, 1990 to 2023.**

| Year | Both sexes (95% UI) | Male | Female |
|---|---|---|---|
| 1990 | 11,196.8 (8,496.6 to 14,392.7) | 10,998.6 | 11,435.2 |
| 1991 | 11,171.8 (8,495.3 to 14,351.5) | 10,959.1 | 11,423.7 |
| 1992 | 11,147.7 (8,453.7 to 14,314.9) | 10,923.8 | 11,409.9 |
| 1993 | 11,121.3 (8,424.6 to 14,308.0) | 10,887.4 | 11,392.8 |
| 1994 | 11,098.8 (8,418.1 to 14,300.4) | 10,858.5 | 11,376.1 |
| 1995 | 11,076.6 (8,368.8 to 14,303.7) | 10,831.0 | 11,358.5 |
| 1996 | 11,050.9 (8,375.5 to 14,247.6) | 10,801.7 | 11,336.0 |
| 1997 | 11,014.7 (8,342.4 to 14,161.9) | 10,762.5 | 11,302.7 |
| 1998 | 10,976.7 (8,301.6 to 14,065.9) | 10,719.7 | 11,269.1 |
| 1999 | 10,921.8 (8,264.0 to 13,994.2) | 10,660.7 | 11,217.7 |
| 2000 | 10,887.2 (8,246.9 to 13,961.8) | 10,617.0 | 11,191.5 |
| 2001 | 10,852.4 (8,225.7 to 13,913.2) | 10,564.8 | 11,173.6 |
| 2002 | 10,797.3 (8,178.0 to 13,823.4) | 10,481.8 | 11,145.6 |
| 2003 | 10,752.5 (8,109.2 to 13,759.6) | 10,404.9 | 11,132.0 |
| 2004 | 10,699.8 (8,077.5 to 13,701.1) | 10,323.2 | 11,108.4 |
| 2005 | 10,672.8 (8,077.1 to 13,671.0) | 10,279.9 | 11,098.2 |
| 2006 | 10,629.4 (8,007.2 to 13,654.4) | 10,227.1 | 11,065.7 |
| 2007 | 10,571.3 (7,960.3 to 13,669.2) | 10,158.9 | 11,019.5 |
| 2008 | 10,532.9 (7,932.7 to 13,584.0) | 10,110.5 | 10,992.5 |
| 2009 | 10,486.7 (7,899.5 to 13,495.2) | 10,058.2 | 10,954.3 |
| 2010 | 10,461.2 (7,890.9 to 13,423.6) | 10,026.6 | 10,936.7 |
| 2011 | 10,453.9 (7,914.6 to 13,421.2) | 10,015.2 | 10,935.5 |
| 2012 | 10,462.3 (7,936.9 to 13,371.3) | 10,016.0 | 10,953.6 |
| 2013 | 10,449.1 (7,943.9 to 13,324.6) | 10,002.2 | 10,943.7 |
| 2014 | 10,456.9 (7,945.2 to 13,347.0) | 10,004.5 | 10,958.9 |
| 2015 | 10,449.3 (7,941.8 to 13,365.9) | 9,993.7 | 10,955.4 |
| 2016 | 10,414.7 (7,920.2 to 13,336.8) | 9,955.7 | 10,924.0 |
| 2017 | 10,376.0 (7,893.4 to 13,355.4) | 9,912.7 | 10,890.0 |
| 2018 | 10,317.8 (7,858.0 to 13,283.7) | 9,855.3 | 10,832.2 |
| 2019 | 10,268.2 (7,830.4 to 13,233.8) | 9,806.4 | 10,782.5 |
| 2020 | 10,673.6 (8,119.7 to 13,923.4) | 10,152.2 | 11,247.4 |
| 2021 | 10,929.6 (8,333.1 to 14,221.4) | 10,385.9 | 11,528.6 |
| 2022 | 10,852.8 (8,276.7 to 14,175.8) | 10,337.8 | 11,428.4 |
| 2023 | 10,734.5 (8,175.8 to 13,950.4) | 10,245.9 | 11,286.9 |

**Table S4. COVID-19 age-standardised rates of years lived with disability and of incidence, Romania, 2020 to 2023.**

| Year | YLD rate (95% UI) | Incidence rate (95% UI) |
|---|---|---|
| 2020 | 286.7 (144.8 to 572.1) | 28,166 (24,689 to 31,514) |
| 2021 | 531.8 (248.6 to 1,065.0) | 54,065 (43,325 to 65,198) |
| 2022 | 422.2 (179.6 to 806.4) | 128,479 (63,250 to 267,037) |
| 2023 | 193.5 (61.5 to 477.8) | 93,588 (16,460 to 258,049) |

**Table S5. Age-standardised rate of years lived with disability from all causes, *eighteen locations*, 1990 and 2023.**

| Location | Rate 1990 (95% UI) | Rate 2023 (95% UI) | 2023 as % of 1990 |
|---|---|---|---|
| Serbia | 10,440.0 (7,961.2 to 13,469.2) | 10,675.8 (8,073.1 to 13,889.0) | 102.3 |
| Albania | 10,993.7 (8,366.5 to 14,206.9) | 10,702.2 (8,073.7 to 13,889.5) | 97.3 |
| Romania | 11,196.8 (8,496.6 to 14,392.7) | 10,734.5 (8,175.8 to 13,950.4) | 95.9 |
| Bulgaria | 10,871.0 (8,276.6 to 13,988.2) | 10,837.9 (8,217.4 to 14,294.8) | 99.7 |
| Slovakia | 11,114.6 (8,447.3 to 14,331.4) | 10,900.1 (8,212.0 to 14,111.1) | 98.1 |
| North Macedonia | 10,589.6 (8,102.2 to 13,572.0) | 10,936.6 (8,421.4 to 14,035.1) | 103.3 |
| Central Europe | 11,213.6 (8,519.6 to 14,414.1) | 10,949.8 (8,315.1 to 14,296.9) | 97.6 |
| Poland | 11,386.0 (8,643.2 to 14,713.8) | 10,984.9 (8,337.0 to 14,401.6) | 96.5 |
| Montenegro | 10,410.9 (7,914.9 to 13,347.3) | 11,077.9 (8,439.1 to 14,373.1) | 106.4 |
| Hungary | 11,780.8 (8,912.8 to 15,262.2) | 11,103.1 (8,450.1 to 14,385.8) | 94.2 |
| Croatia | 10,909.2 (8,374.5 to 14,026.6) | 11,114.9 (8,472.2 to 14,431.8) | 101.9 |
| Slovenia | 11,108.4 (8,436.6 to 14,263.6) | 11,160.8 (8,452.3 to 14,396.2) | 100.5 |
| Central Europe, Eastern Europe, and Central Asia | 11,413.0 (8,642.7 to 14,630.7) | 11,184.3 (8,491.4 to 14,511.8) | 98.0 |
| Czechia | 11,432.8 (8,661.2 to 14,639.7) | 11,213.0 (8,548.1 to 14,647.9) | 98.1 |
| Bosnia and Herzegovina | 11,055.2 (8,425.1 to 14,273.3) | 11,382.3 (8,629.4 to 14,878.7) | 103.0 |
| Western Europe | 10,657.7 (8,065.9 to 13,543.3) | 11,426.6 (8,678.3 to 14,731.7) | 107.2 |
| Eastern Europe | 11,563.5 (8,734.5 to 14,789.1) | 11,492.2 (8,760.6 to 14,842.6) | 99.4 |
| Global | 11,767.5 (8,952.8 to 15,033.3) | 11,679.1 (8,906.2 to 15,092.5) | 99.2 |

**Table S6. Percentage change in the four headline measures, *eighteen locations*, 1990 to 2023.**

| Location | YLD rate | Attributable fraction | Summary exposure value | HALE at birth |
|---|---|---|---|---|
| North Macedonia | +3.3 (-2.6 to 9.3) | -3.2 (-12.5 to 4.7) | +8.4 (-0.2 to 18.5) | +6.9 (6.0 to 7.7) |
| Slovenia | +0.5 (-4.3 to 5.1) | -0.7 (-9.5 to 8.2) | +5.7 (-5.9 to 20.7) | +9.3 (8.4 to 10.1) |
| Eastern Europe | -0.6 (-4.5 to 3.6) | -7.0 (-14.8 to 0.4) | +0.6 (-6.3 to 7.5) | +4.5 (3.7 to 5.4) |
| Montenegro | +6.4 (0.7 to 12.1) | -0.0 (-9.5 to 8.4) | +3.3 (-4.9 to 13.1) | +3.9 (2.8 to 4.7) |
| Poland | -3.5 (-8.5 to 1.4) | -7.5 (-14.3 to 0.4) | +0.1 (-6.9 to 8.3) | +9.4 (8.6 to 10.2) |
| Global | -0.8 (-4.8 to 3.6) | -6.4 (-11.7 to -0.7) | -3.8 (-7.4 to -0.5) | +13.0 (12.2 to 13.7) |
| Romania | -4.1 (-10.1 to 1.3) | -9.4 (-16.8 to -0.7) | +3.8 (-3.9 to 11.9) | +8.5 (7.7 to 9.4) |
| Central Europe | -2.4 (-7.5 to 2.3) | -7.9 (-14.9 to 0.2) | +1.3 (-3.5 to 6.6) | +8.8 (8.0 to 9.5) |
| Western Europe | +7.2 (3.2 to 11.8) | -8.6 (-15.5 to -1.8) | +2.5 (-2.3 to 7.1) | +5.4 (4.5 to 6.1) |
| Bosnia and Herzegovina | +3.0 (-3.1 to 9.6) | -8.2 (-17.0 to 0.7) | +3.1 (-4.5 to 12.0) | +5.8 (4.7 to 6.9) |
| Serbia | +2.3 (-3.6 to 8.0) | -7.0 (-15.7 to 1.7) | +3.9 (-5.6 to 12.9) | +7.2 (6.1 to 8.3) |
| Central Europe, Eastern Europe, and Central Asia | -2.0 (-6.8 to 2.6) | -6.6 (-13.9 to 1.0) | +0.7 (-4.8 to 6.0) | +6.5 (5.8 to 7.2) |
| Slovakia | -1.9 (-7.7 to 3.5) | -8.3 (-16.5 to 0.0) | -0.9 (-9.2 to 8.6) | +8.9 (7.9 to 9.7) |
| Bulgaria | -0.3 (-5.6 to 5.3) | -2.3 (-10.9 to 6.7) | +6.7 (-1.3 to 14.9) | +5.7 (4.8 to 6.4) |
| Croatia | +1.9 (-3.7 to 8.2) | -0.6 (-8.7 to 7.9) | +7.9 (-1.7 to 16.9) | +6.7 (5.7 to 7.5) |
| Albania | -2.7 (-8.5 to 3.9) | -13.9 (-22.7 to -4.7) | +2.0 (-6.9 to 9.9) | +10.0 (8.8 to 11.3) |
| Hungary | -5.8 (-10.9 to -0.4) | -11.0 (-18.7 to -3.3) | -4.5 (-11.1 to 2.0) | +10.2 (9.2 to 11.0) |
| Czechia | -1.9 (-7.3 to 3.2) | -11.5 (-17.7 to -5.5) | -3.1 (-9.6 to 3.3) | +10.0 (9.0 to 10.7) |

*Table S7. Age-standardised rate of years lived with disability per 100 000 population by level 2 cause, Romania, 1990 and 2023. This table provides the ranks and uncertainty intervals underlying Figure 2 in the manuscript.*

| Cause | Rank 1990 | Rate 1990 (95% UI) | Rank 2023 | Rate 2023 (95% UI) | 2023 as % of 1990 |
|---|---|---|---|---|---|
| Musculoskeletal disorders | 1 | 2,091.8 (1,528.2 to 2,866.0) | 1 | 2,080.8 (1,543.6 to 2,848.9) | 99.5 |
| Mental disorders | 3 | 1,518.4 (1,095.1 to 1,915.8) | 2 | 1,768.1 (1,279.3 to 2,373.8) | 116.4 |
| Unintentional injuries | 2 | 1,561.1 (1,146.5 to 2,120.8) | 3 | 1,168.3 (816.2 to 1,605.2) | 74.8 |
| Other non-communicable diseases | 4 | 874.8 (596.4 to 1,255.3) | 4 | 832.0 (580.1 to 1,161.3) | 95.1 |
| Neurological disorders | 5 | 790.3 (530.4 to 1,102.5) | 5 | 788.8 (517.3 to 1,092.3) | 99.8 |
| Sense organ diseases | 6 | 744.0 (526.3 to 1,007.7) | 6 | 715.7 (502.3 to 975.3) | 96.2 |
| Cardiovascular diseases | 7 | 548.9 (399.4 to 703.2) | 7 | 409.7 (304.4 to 534.3) | 74.6 |
| Diabetes and kidney diseases | 14 | 261.9 (188.2 to 349.9) | 8 | 400.8 (293.3 to 542.7) | 153.0 |
| Chronic respiratory diseases | 9 | 404.5 (317.2 to 503.0) | 9 | 391.5 (303.1 to 479.0) | 96.8 |
| Maternal and neonatal disorders | 10 | 330.5 (232.3 to 450.1) | 10 | 362.3 (262.2 to 473.5) | 109.6 |
| Skin and subcutaneous diseases | 13 | 281.4 (192.2 to 396.0) | 11 | 313.2 (214.8 to 435.7) | 111.3 |
| Nutritional deficiencies | 8 | 498.1 (231.4 to 1,010.8) | 12 | 292.0 (153.1 to 569.2) | 58.6 |
| Digestive diseases | 11 | 321.1 (234.8 to 443.8) | 13 | 290.1 (212.6 to 401.3) | 90.3 |
| Respiratory infections and tuberculosis | 16 | 111.5 (77.1 to 154.3) | 14 | 277.7 (136.2 to 572.1) | 249.1 |
| Substance use disorders | 12 | 304.3 (207.5 to 416.2) | 15 | 245.2 (171.0 to 344.8) | 80.6 |
| Transport injuries | 15 | 220.0 (163.0 to 287.1) | 16 | 112.5 (81.4 to 150.4) | 51.1 |
| Neoplasms | 19 | 71.1 (51.0 to 93.0) | 17 | 101.7 (73.6 to 139.7) | 143.0 |
| Self-harm and interpersonal violence | 17 | 93.2 (72.4 to 118.4) | 18 | 68.8 (52.6 to 87.5) | 73.8 |
| Neglected tropical diseases and malaria | 18 | 78.5 (46.6 to 128.4) | 19 | 47.1 (28.3 to 80.3) | 60.0 |
| Other infectious diseases | 20 | 54.0 (32.0 to 91.9) | 20 | 34.9 (22.2 to 56.4) | 64.7 |
| Enteric infections | 21 | 28.6 (18.8 to 40.0) | 21 | 18.0 (12.0 to 25.3) | 63.2 |
| HIV/AIDS and sexually transmitted infections | 22 | 9.1 (5.3 to 15.4) | 22 | 15.4 (9.7 to 23.3) | 169.4 |

**Table S8. Percentage change in the age-standardised rate of years lived with disability by level 2 cause, Romania, 1990 to 2023.**

| Cause | Percentage change 1990 to 2023 (95% UI) | Direction |
|---|---|---|
| All causes | -4.1 (-10.1 to 1.3) | no change |
| Musculoskeletal disorders | -0.5 (-4.0 to 2.5) | no change |
| Mental disorders | +16.4 (2.4 to 35.6) | increase |
| Unintentional injuries | -25.2 (-29.7 to -21.7) | decrease |
| Other non-communicable diseases | -4.9 (-10.2 to 0.9) | no change |
| Neurological disorders | -0.2 (-17.9 to 24.7) | no change |
| Sense organ diseases | -3.8 (-6.3 to -0.9) | decrease |
| Cardiovascular diseases | -25.4 (-28.8 to -22.0) | decrease |
| Diabetes and kidney diseases | +53.0 (42.0 to 65.4) | increase |
| Chronic respiratory diseases | -3.2 (-9.9 to 4.3) | no change |
| Maternal and neonatal disorders | +9.6 (-12.2 to 42.2) | no change |
| Skin and subcutaneous diseases | +11.3 (7.3 to 15.8) | increase |
| Nutritional deficiencies | -41.4 (-74.8 to 35.2) | no change |
| Digestive diseases | -9.7 (-14.0 to -5.3) | decrease |
| Respiratory infections and tuberculosis | +149.1 (34.3 to 380.7) | increase |
| Substance use disorders | -19.4 (-29.9 to -8.6) | decrease |
| Transport injuries | -48.9 (-52.2 to -46.5) | decrease |
| Neoplasms | +43.0 (20.0 to 72.8) | increase |
| Self-harm and interpersonal violence | -26.2 (-30.8 to -21.7) | decrease |
| Neglected tropical diseases and malaria | -40.0 (-65.9 to 3.3) | no change |
| Other infectious diseases | -35.3 (-61.3 to 5.3) | no change |
| Enteric infections | -36.8 (-42.9 to -30.7) | decrease |
| HIV/AIDS and sexually transmitted infections | +69.4 (39.1 to 133.6) | increase |

**Table S9. Age-standardised rate of years lived with disability by level 3 cause, Romania, 1990 and 2023.**

| Cause | Rank 2023 | Rate 1990 (95% UI) | Rate 2023 (95% UI) |
|---|---|---|---|
| Low back pain | 1 | 1,526.3 (1,095.6 to 2,054.6) | 1,432.1 (1,016.9 to 1,930.3) |
| Anxiety disorders | 2 | 486.1 (309.7 to 720.4) | 688.8 (425.3 to 1,033.6) |
| Falls | 3 | 695.8 (508.8 to 936.9) | 610.1 (436.8 to 832.2) |
| Age-related and other hearing loss | 4 | 555.6 (391.5 to 744.1) | 551.0 (386.9 to 740.3) |
| Headache disorders | 5 | 537.3 (366.5 to 731.5) | 536.1 (369.5 to 733.1) |
| Neonatal disorders | 6 | 324.1 (226.7 to 442.3) | 356.5 (257.2 to 466.3) |
| Depressive disorders | 7 | 324.1 (220.6 to 451.4) | 340.9 (235.6 to 477.8) |
| Exposure to mechanical forces | 8 | 463.0 (327.7 to 664.1) | 338.7 (221.7 to 498.7) |
| Diabetes mellitus | 9 | 197.8 (134.0 to 270.4) | 306.5 (211.5 to 424.8) |
| Oral disorders | 10 | 320.8 (201.1 to 474.2) | 293.9 (181.6 to 435.1) |
| Dietary iron deficiency | 11 | 477.9 (214.8 to 982.7) | 286.1 (147.6 to 560.9) |
| Gynecological diseases | 12 | 243.1 (160.4 to 338.9) | 235.2 (157.0 to 329.9) |
| Neck pain | 13 | 231.8 (153.7 to 342.0) | 229.9 (154.8 to 336.0) |
| Osteoarthritis | 14 | 207.0 (97.1 to 456.1) | 228.9 (107.7 to 498.5) |
| Chronic obstructive pulmonary disease | 15 | 231.9 (182.9 to 278.7) | 212.8 (171.4 to 252.2) |
| Alcohol use disorders | 16 | 258.9 (179.5 to 360.3) | 197.4 (136.1 to 279.9) |
| COVID-19 | 17 | *Not applicable* | 193.5 (61.5 to 477.8) |
| Schizophrenia | 18 | 185.9 (130.8 to 238.7) | 187.1 (131.3 to 244.7) |
| Stroke | 19 | 255.5 (179.2 to 325.9) | 173.6 (124.5 to 222.1) |
| Asthma | 20 | 158.9 (97.0 to 230.5) | 163.4 (100.6 to 238.2) |
| Other musculoskeletal disorders | 21 | 105.3 (67.1 to 154.9) | 160.7 (105.5 to 228.0) |
| Blindness and vision loss | 22 | 176.0 (113.9 to 262.7) | 151.7 (94.8 to 233.9) |
| Endocrine, metabolic, blood, and immune disorders | 23 | 138.2 (90.4 to 203.5) | 147.1 (98.4 to 212.3) |
| Gallbladder and biliary diseases | 24 | 145.6 (94.9 to 203.7) | 125.0 (78.3 to 176.0) |
| Dermatitis | 25 | 124.0 (76.3 to 190.2) | 123.9 (76.2 to 189.5) |
| Autism spectrum disorders | 26 | 100.3 (39.5 to 206.0) | 122.2 (54.2 to 250.9) |
| Bipolar disorder | 27 | 119.2 (70.3 to 176.9) | 119.8 (68.9 to 173.4) |
| Alzheimer's disease and other dementias | 28 | 114.4 (79.4 to 148.0) | 115.0 (79.9 to 149.9) |

| Cause | Rank 2023 | Rate 1990 (95% UI) | Rate 2023 (95% UI) |
|---|---|---|---|
| Ischemic heart disease | 29 | 169.6 (120.4 to 230.5) | 114.3 (79.2 to 157.2) |
| Other mental disorders | 30 | 111.0 (72.1 to 158.7) | 111.8 (73.2 to 158.9) |
| Upper digestive system diseases | 31 | 99.3 (55.1 to 168.8) | 99.9 (56.3 to 169.1) |
| Road injuries | 32 | 199.3 (147.7 to 261.8) | 98.8 (71.1 to 131.9) |
| Chronic kidney disease | 33 | 64.1 (46.0 to 84.0) | 94.2 (67.8 to 124.0) |
| Idiopathic epilepsy | 34 | 112.9 (26.6 to 247.0) | 91.6 (14.4 to 209.0) |
| Other unintentional injuries | 35 | 160.1 (109.7 to 228.8) | 87.4 (59.0 to 129.6) |
| Congenital birth defects | 36 | 84.6 (59.9 to 117.0) | 86.4 (60.4 to 115.8) |
| Urticaria | 37 | 70.3 (46.6 to 99.5) | 71.9 (47.4 to 102.9) |
| Conduct disorder | 38 | 68.6 (39.3 to 110.2) | 69.0 (38.6 to 108.8) |
| Fire, heat, and hot substances | 39 | 129.8 (94.4 to 174.4) | 65.9 (41.8 to 100.3) |
| Interpersonal violence | 40 | 77.1 (59.4 to 97.8) | 59.6 (45.2 to 75.9) |
| Idiopathic developmental intellectual disability | 41 | 59.6 (29.3 to 111.0) | 52.0 (24.4 to 96.2) |
| Atrial fibrillation and flutter | 42 | 52.7 (33.2 to 80.7) | 51.7 (32.6 to 78.4) |
| Eating disorders | 43 | 38.9 (21.7 to 59.6) | 51.7 (29.9 to 77.5) |
| Drug use disorders | 44 | 45.3 (32.0 to 58.0) | 47.8 (34.1 to 62.0) |
| Upper respiratory infections | 45 | 46.6 (28.9 to 69.2) | 46.3 (28.8 to 69.3) |
| Urinary diseases and male infertility | 46 | 41.5 (27.0 to 59.5) | 42.3 (27.6 to 59.9) |
| Psoriasis | 47 | 29.5 (20.8 to 38.8) | 41.1 (29.8 to 55.4) |
| Other cardiovascular and circulatory diseases | 48 | 32.8 (21.3 to 47.6) | 29.0 (18.3 to 43.5) |
| Hemoglobinopathies and hemolytic anemias | 49 | 46.6 (20.8 to 96.2) | 27.2 (13.7 to 53.8) |
| Acne vulgaris | 50 | 10.5 (6.3 to 16.6) | 26.6 (16.4 to 41.2) |
| Other neglected tropical diseases | 51 | 42.4 (19.9 to 85.0) | 26.1 (14.5 to 50.5) |
| Other neurological disorders | 52 | 10.9 (4.5 to 20.7) | 25.8 (11.3 to 47.6) |
| Attention-deficit/hyperactivity disorder | 53 | 24.6 (14.2 to 37.4) | 24.8 (14.1 to 37.3) |
| Otitis media | 54 | 27.8 (16.3 to 44.7) | 23.6 (14.1 to 39.5) |
| Cirrhosis and other chronic liver diseases | 55 | 26.8 (19.3 to 36.7) | 23.4 (16.4 to 32.0) |

| Cause | Rank 2023 | Rate 1990 (95% UI) | Rate 2023 (95% UI) |
|---|---|---|---|
| Other unspecified infectious diseases | 56 | 34.8 (16.3 to 70.0) | 22.5 (12.7 to 42.2) |
| Cysticercosis | 57 | 35.2 (18.1 to 58.3) | 20.2 (9.1 to 38.7) |
| Breast cancer | 58 | 13.5 (9.7 to 18.6) | 19.3 (13.6 to 27.2) |
| Rheumatoid arthritis | 59 | 11.8 (7.7 to 17.5) | 18.0 (12.2 to 26.6) |
| Diarrheal diseases | 60 | 28.5 (18.8 to 39.9) | 18.0 (12.0 to 25.3) |
| Foreign body | 61 | 23.4 (16.7 to 31.8) | 17.1 (11.4 to 24.3) |
| Poisonings | 62 | 43.1 (28.0 to 62.0) | 16.8 (10.8 to 24.1) |
| Other skin and subcutaneous diseases | 63 | 13.0 (6.2 to 23.1) | 15.8 (7.7 to 28.3) |
| Environmental heat and cold exposure | 64 | 20.6 (15.0 to 28.0) | 15.7 (10.9 to 22.5) |
| Viral skin diseases | 65 | 15.1 (9.9 to 23.0) | 15.5 (10.0 to 23.1) |
| Inguinal, femoral, and abdominal hernia | 66 | 23.5 (16.4 to 31.9) | 15.3 (10.1 to 22.6) |
| Parkinson's disease | 67 | 9.5 (6.7 to 13.0) | 14.8 (10.5 to 19.9) |
| Other transport injuries | 68 | 20.7 (15.3 to 27.7) | 13.7 (10.0 to 18.5) |
| Colon and rectum cancer | 69 | 6.5 (4.6 to 8.7) | 13.4 (9.7 to 17.8) |
| Other sense organ diseases | 70 | 12.3 (7.7 to 18.4) | 12.9 (8.1 to 18.9) |
| Tuberculosis | 71 | 33.7 (22.8 to 46.5) | 12.8 (8.4 to 18.1) |
| Non-rheumatic valvular heart disease | 72 | 9.1 (5.1 to 15.5) | 12.1 (6.8 to 20.1) |
| Animal contact | 73 | 16.2 (11.2 to 23.2) | 11.5 (7.8 to 16.8) |
| Gout | 74 | 9.6 (6.6 to 13.8) | 11.2 (7.4 to 16.5) |
| Hypertensive heart disease | 75 | 9.3 (5.2 to 14.1) | 11.1 (6.0 to 16.2) |
| Other chronic respiratory diseases | 76 | 7.9 (6.1 to 9.6) | 10.5 (8.1 to 13.1) |
| Other digestive diseases | 77 | 10.3 (7.4 to 13.9) | 9.4 (6.8 to 12.9) |
| Cardiomyopathy and myocarditis | 78 | 8.3 (5.5 to 11.5) | 8.8 (5.7 to 13.1) |
| Prostate cancer | 79 | 3.3 (2.3 to 4.8) | 8.7 (6.0 to 11.8) |
| Sexually transmitted infections excluding HIV | 80 | 8.3 (4.6 to 14.6) | 8.4 (4.5 to 14.4) |

| Cause | Rank 2023 | Rate 1990 (95% UI) | Rate 2023 (95% UI) |
|---|---|---|---|
| Fungal skin diseases | 81 | 8.5 (3.5 to 17.3) | 7.8 (3.1 to 16.2) |
| Pancreatitis | 82 | 6.9 (3.3 to 12.1) | 7.3 (3.8 to 12.7) |
| Self-harm | 83 | 10.3 (7.5 to 13.5) | 7.1 (5.1 to 9.6) |
| HIV/AIDS | 84 | 0.8 (0.5 to 1.1) | 7.0 (4.1 to 11.2) |
| Tracheal, bronchus, and lung cancer | 85 | 6.0 (4.1 to 7.7) | 6.7 (4.8 to 8.8) |
| Maternal disorders | 86 | 6.4 (3.6 to 10.2) | 5.8 (3.3 to 8.8) |
| Acute hepatitis | 87 | 6.5 (4.1 to 9.4) | 5.6 (3.6 to 8.2) |
| Lower extremity peripheral arterial disease | 88 | 6.0 (2.7 to 11.5) | 5.5 (2.4 to 10.7) |
| Cervical cancer | 89 | 6.1 (4.1 to 8.6) | 5.4 (3.7 to 7.8) |
| Multiple sclerosis | 90 | 4.8 (3.5 to 6.4) | 4.8 (3.5 to 6.5) |
| Bladder cancer | 91 | 2.9 (2.0 to 4.0) | 4.7 (3.2 to 6.3) |
| Other neoplasms | 92 | 2.8 (1.8 to 4.3) | 4.5 (2.9 to 6.9) |
| Interstitial lung disease and pulmonary sarcoidosis | 93 | 5.5 (3.7 to 7.9) | 4.3 (2.8 to 5.9) |
| Iodine deficiency | 94 | 5.1 (2.4 to 9.4) | 4.2 (1.8 to 7.6) |
| Appendicitis | 95 | 4.1 (2.4 to 6.5) | 4.1 (2.4 to 6.5) |
| Uterine cancer | 96 | 2.6 (1.8 to 3.5) | 3.6 (2.5 to 5.1) |
| Rheumatic heart disease | 97 | 5.3 (3.5 to 7.7) | 3.3 (2.2 to 4.8) |
| Scabies | 98 | 3.4 (1.9 to 5.5) | 3.3 (1.9 to 5.5) |
| Leukemia | 99 | 2.4 (1.6 to 3.4) | 2.9 (1.9 to 4.4) |
| Inflammatory bowel disease | 100 | 2.7 (1.7 to 4.0) | 2.8 (1.9 to 4.0) |
| Non-Hodgkin lymphoma | 101 | 1.2 (0.7 to 2.1) | 2.8 (1.5 to 5.2) |
| Decubitus ulcer | 102 | 2.6 (1.7 to 3.4) | 2.5 (1.7 to 3.2) |
| Larynx cancer | 103 | 2.0 (1.4 to 2.7) | 2.4 (1.7 to 3.3) |
| Kidney cancer | 104 | 1.1 (0.8 to 1.5) | 2.4 (1.6 to 3.3) |
| Stomach cancer | 105 | 3.4 (2.4 to 4.5) | 2.4 (1.7 to 3.2) |
| Ovarian cancer | 106 | 2.2 (1.5 to 3.1) | 2.4 (1.5 to 3.4) |
| Lip and oral cavity cancer | 107 | 1.2 (0.8 to 1.6) | 2.2 (1.4 to 3.1) |
| Other malignant neoplasms | 108 | 1.8 (1.3 to 2.6) | 2.2 (1.5 to 3.1) |
| Malignant skin melanoma | 109 | 0.8 (0.5 to 1.2) | 2.1 (1.3 to 3.2) |

| Cause | Rank 2023 | Rate 1990 (95% UI) | Rate 2023 (95% UI) |
|---|---|---|---|
| Adverse effects of medical treatment | 110 | 1.9 (1.2 to 2.9) | 2.1 (1.4 to 3.3) |
| Conflict and terrorism | 111 | 5.8 (4.0 to 8.7) | 2.1 (1.4 to 3.2) |
| Pancreatic cancer | 112 | 1.3 (0.9 to 1.8) | 1.9 (1.2 to 2.4) |
| Brain and central nervous system cancer | 113 | 1.6 (1.1 to 2.0) | 1.9 (1.3 to 2.6) |
| Meningitis | 114 | 6.7 (4.8 to 9.1) | 1.8 (1.3 to 2.5) |
| Encephalitis | 115 | 3.3 (2.3 to 4.4) | 1.8 (1.3 to 2.5) |
| Alopecia areata | 116 | 1.8 (1.1 to 2.7) | 1.8 (1.2 to 2.6) |
| Pruritus | 117 | 1.3 (0.6 to 2.6) | 1.7 (0.8 to 3.4) |
| Pertussis | 118 | 1.1 (0.6 to 1.8) | 1.6 (0.9 to 2.7) |
| Lower respiratory infections | 119 | 3.4 (2.3 to 4.7) | 1.6 (1.0 to 2.2) |
| Varicella and herpes zoster | 120 | 1.5 (0.8 to 2.3) | 1.5 (0.8 to 2.3) |
| Drowning | 121 | 3.5 (2.5 to 4.7) | 1.5 (1.0 to 2.0) |
| Testicular cancer | 122 | 0.6 (0.3 to 1.1) | 1.5 (0.8 to 2.4) |
| Vitamin A deficiency | 123 | 12.8 (5.4 to 31.1) | 1.4 (0.5 to 3.1) |
| Paralytic ileus and intestinal obstruction | 124 | 1.1 (0.8 to 1.5) | 1.4 (0.9 to 1.9) |
| Vascular intestinal disorders | 125 | 0.9 (0.5 to 1.2) | 1.4 (0.9 to 2.0) |
| Electrocution | 126 | 3.7 (2.6 to 5.1) | 1.4 (0.9 to 2.0) |
| Bacterial skin diseases | 127 | 1.3 (0.7 to 2.3) | 1.3 (0.7 to 2.2) |
| Malignant neoplasm of bone and articular cartilage | 128 | 2.4 (1.6 to 3.6) | 1.1 (0.7 to 1.7) |
| Thyroid cancer | 129 | 1.0 (0.6 to 1.6) | 1.1 (0.7 to 1.8) |
| Other pharynx cancer | 130 | 0.4 (0.3 to 0.5) | 1.1 (0.7 to 1.5) |
| Soft tissue and other extraosseous sarcomas | 131 | 0.7 (0.5 to 0.9) | 0.9 (0.6 to 1.3) |
| Cystic echinococcosis | 132 | 0.9 (0.6 to 1.3) | 0.8 (0.5 to 1.2) |
| Motor neuron disease | 133 | 0.7 (0.5 to 0.9) | 0.7 (0.5 to 1.0) |
| Multiple myeloma | 134 | 0.4 (0.3 to 0.6) | 0.7 (0.4 to 1.4) |
| Esophageal cancer | 135 | 0.5 (0.3 to 0.6) | 0.7 (0.5 to 1.0) |
| Liver cancer | 136 | 0.3 (0.2 to 0.5) | 0.7 (0.5 to 0.9) |
| Hodgkin lymphoma | 137 | 0.7 (0.5 to 1.0) | 0.6 (0.4 to 0.9) |

| Cause | Rank 2023 | Rate 1990 (95% UI) | Rate 2023 (95% UI) |
|---|---|---|---|
| Nasopharynx cancer | 138 | 0.2 (0.1 to 0.3) | 0.5 (0.3 to 0.8) |
| Pneumoconiosis | 139 | 0.3 (0.2 to 0.5) | 0.4 (0.3 to 0.7) |
| Gallbladder and biliary tract cancer | 140 | 0.5 (0.3 to 0.7) | 0.3 (0.2 to 0.5) |
| Non-melanoma skin cancer | 141 | 0.3 (0.2 to 0.5) | 0.3 (0.2 to 0.5) |
| Protein-energy malnutrition | 142 | 2.0 (0.9 to 4.1) | 0.2 (0.1 to 0.5) |
| Exposure to forces of nature | 143 | 0.0 (0.0 to 0.1) | 0.2 (0.2 to 0.3) |
| Endocarditis | 144 | 0.1 (0.1 to 0.1) | 0.2 (0.1 to 0.3) |
| Pulmonary Arterial Hypertension | 145 | 0.2 (0.1 to 0.3) | 0.2 (0.1 to 0.3) |
| Eye cancer | 146 | 0.2 (0.1 to 0.3) | 0.1 (0.1 to 0.2) |
| Mesothelioma | 147 | 0.0 (0.0 to 0.1) | 0.1 (0.0 to 0.1) |
| Measles | 148 | 0.1 (0.0 to 0.1) | 0.0 (0.0 to 0.1) |
| Police conflict and executions | 149 | 0.0 (0.0 to 0.0) | 0.0 (0.0 to 0.1) |
| Neuroblastoma and other peripheral nervous cell tumors | 150 | 0.0 (0.0 to 0.1) | 0.0 (0.0 to 0.0) |
| Acute glomerulonephritis | 151 | 0.0 (0.0 to 0.0) | 0.0 (0.0 to 0.0) |
| Other intestinal infectious diseases | 152 | 0.0 (0.0 to 0.0) | 0.0 (0.0 to 0.0) |
| Other nutritional deficiencies | 153 | 0.2 (0.0 to 0.7) | 0.0 (0.0 to 0.0) |
| Leishmaniasis | 154 | 0.0 (0.0 to 0.0) | 0.0 (0.0 to 0.0) |
| Typhoid and paratyphoid | 155 | 0.0 (0.0 to 0.0) | 0.0 (0.0 to 0.0) |
| Tetanus | 156 | 0.0 (0.0 to 0.0) | 0.0 (0.0 to 0.0) |
| Invasive Non-typhoidal Salmonella (iNTS) | 157 | 0.0 (0.0 to 0.0) | 0.0 (0.0 to 0.0) |
| Diphtheria | 158 | 0.0 (0.0 to 0.0) | 0.0 (0.0 to 0.0) |
| Rabies | 159 | 0.0 (0.0 to 0.0) | 0.0 (0.0 to 0.0) |
| Dengue | 167 | 0.0 (0.0 to 0.0) | 0.0 (0.0 to 0.0) |
| African trypanosomiasis | 167 | 0.0 (0.0 to 0.0) | 0.0 (0.0 to 0.0) |
| Chagas disease | 167 | 0.0 (0.0 to 0.0) | 0.0 (0.0 to 0.0) |
| Food-borne trematodiases | 167 | 0.0 (0.0 to 0.0) | 0.0 (0.0 to 0.0) |
| Ebola | 167 | 0.0 (0.0 to 0.0) | 0.0 (0.0 to 0.0) |
| Guinea worm disease | 167 | 0.0 (0.0 to 0.0) | 0.0 (0.0 to 0.0) |

| Cause | Rank 2023 | Rate 1990 (95% UI) | Rate 2023 (95% UI) |
|---|---|---|---|
| Intestinal nematode infections | 167 | 0.0 (0.0 to 0.0) | 0.0 (0.0 to 0.0) |
| Onchocerciasis | 167 | 0.0 (0.0 to 0.0) | 0.0 (0.0 to 0.0) |
| Leprosy | 167 | 0.0 (0.0 to 0.0) | 0.0 (0.0 to 0.0) |
| Lymphatic filariasis | 167 | 0.0 (0.0 to 0.0) | 0.0 (0.0 to 0.0) |
| Malaria | 167 | 0.0 (0.0 to 0.0) | 0.0 (0.0 to 0.0) |
| Schistosomiasis | 167 | 0.0 (0.0 to 0.0) | 0.0 (0.0 to 0.0) |
| Trachoma | 167 | 0.0 (0.0 to 0.0) | 0.0 (0.0 to 0.0) |
| Yellow fever | 167 | 0.0 (0.0 to 0.0) | 0.0 (0.0 to 0.0) |
| Zika virus | 167 | 0.0 (0.0 to 0.0) | 0.0 (0.0 to 0.0) |

**Table S10. Twenty-five largest level 3 causes by sex, age-standardised rate, Romania, 2023.**

| Cause | Male | Female | Female as % of male |
|---|---|---|---|
| Low back pain | 1,216.5 | 1,633.6 | 134.3 |
| Anxiety disorders | 475.3 | 902.6 | 189.9 |
| Falls | 771.7 | 464.0 | 60.1 |
| Age-related and other hearing loss | 574.4 | 527.4 | 91.8 |
| Headache disorders | 336.6 | 737.9 | 219.3 |
| Neonatal disorders | 370.9 | 340.4 | 91.8 |
| Depressive disorders | 299.6 | 380.2 | 126.9 |
| Exposure to mechanical forces | 542.2 | 147.0 | 27.1 |
| Diabetes mellitus | 365.3 | 257.0 | 70.4 |
| Oral disorders | 278.4 | 305.7 | 109.8 |
| Dietary iron deficiency | 208.4 | 365.5 | 175.4 |
| Gynecological diseases | 0.0 | 476.1 | |
| Neck pain | 206.4 | 252.6 | 122.3 |
| Osteoarthritis | 195.1 | 255.8 | 131.1 |
| Chronic obstructive pulmonary disease | 250.4 | 186.2 | 74.4 |
| Alcohol use disorders | 246.0 | 155.5 | 63.2 |
| COVID-19 | 171.9 | 214.5 | 124.8 |
| Schizophrenia | 195.3 | 178.8 | 91.6 |
| Stroke | 204.7 | 155.2 | 75.8 |
| Asthma | 139.1 | 186.4 | 134.1 |
| Other musculoskeletal disorders | 48.6 | 264.0 | 542.8 |
| Blindness and vision loss | 141.2 | 159.9 | 113.3 |
| Endocrine, metabolic, blood, and immune disorders | 96.4 | 196.6 | 204.0 |
| Gallbladder and biliary diseases | 75.3 | 172.8 | 229.6 |
| Dermatitis | 106.4 | 141.8 | 133.3 |

**Table S11. Percentage change in the age-standardised rate of years lived with disability by level 3 cause, Romania, 1990 to 2023.**

| Cause | Rate 2023 | Percentage change 1990 to 2023 (95% UI) | Direction |
|---|---|---|---|
| Low back pain | 1,432.1 | -6.2 (-10.2 to -2.3) | decrease |
| Anxiety disorders | 688.8 | +41.7 (4.5 to 101.8) | increase |
| Falls | 610.1 | -12.3 (-17.4 to -8.4) | decrease |
| Age-related and other hearing loss | 551.0 | -0.8 (-3.5 to 2.0) | no change |
| Headache disorders | 536.1 | -0.2 (-2.9 to 2.3) | no change |
| Neonatal disorders | 356.5 | +10.0 (-12.4 to 44.3) | no change |
| Depressive disorders | 340.9 | +5.2 (-12.2 to 30.5) | no change |
| Exposure to mechanical forces | 338.7 | -26.9 (-31.4 to -23.2) | decrease |
| Diabetes mellitus | 306.5 | +55.0 (43.4 to 67.8) | increase |
| Oral disorders | 293.9 | -8.4 (-15.6 to 0.4) | no change |
| Dietary iron deficiency | 286.1 | -40.1 (-74.6 to 41.5) | no change |
| Gynecological diseases | 235.2 | -3.3 (-7.0 to 0.4) | no change |
| Neck pain | 229.9 | -0.8 (-3.4 to 2.2) | no change |
| Osteoarthritis | 228.9 | +10.6 (6.9 to 14.0) | increase |
| Chronic obstructive pulmonary disease | 212.8 | -8.2 (-19.4 to 4.2) | no change |
| Alcohol use disorders | 197.4 | -23.8 (-35.0 to -11.8) | decrease |
| COVID-19 | 193.5 | *Not estimable (1990 rate = 0); absolute change +193.5 (61.5 to 477.8)* | *increase on absolute scale* |
| Schizophrenia | 187.1 | +0.6 (-7.2 to 7.7) | no change |
| Stroke | 173.6 | -32.1 (-36.1 to -27.2) | decrease |
| Asthma | 163.4 | +2.9 (-2.2 to 8.9) | no change |
| Other musculoskeletal disorders | 160.7 | +52.6 (33.5 to 75.8) | increase |
| Blindness and vision loss | 151.7 | -13.8 (-19.3 to -7.8) | decrease |
| Endocrine, metabolic, blood, and immune disorders | 147.1 | +6.4 (-5.1 to 18.6) | no change |
| Gallbladder and biliary diseases | 125.0 | -14.1 (-21.0 to -6.6) | decrease |
| Dermatitis | 123.9 | -0.1 (-3.7 to 3.3) | no change |
| Autism spectrum disorders | 122.2 | +21.8 (-36.9 to 169.0) | no change |

| Cause | Rate 2023 | Percentage change 1990 to 2023 (95% UI) | Direction |
|---|---|---|---|
| Bipolar disorder | 119.8 | +0.4 (-5.4 to 6.9) | no change |
| Alzheimer's disease and other dementias | 115.0 | +0.5 (-4.4 to 5.3) | no change |
| Ischemic heart disease | 114.3 | -32.6 (-38.3 to -26.2) | decrease |
| Other mental disorders | 111.8 | +0.8 (-4.4 to 5.6) | no change |
| Upper digestive system diseases | 99.9 | +0.6 (-2.6 to 3.9) | no change |
| Road injuries | 98.8 | -50.4 (-53.6 to -48.3) | decrease |
| Chronic kidney disease | 94.2 | +47.0 (19.0 to 76.1) | increase |
| Idiopathic epilepsy | 91.6 | -18.8 (-83.5 to 463.4) | no change |
| Other unintentional injuries | 87.4 | -45.4 (-48.3 to -42.5) | decrease |
| Congenital birth defects | 86.4 | +2.1 (-3.8 to 8.5) | no change |
| Urticaria | 71.9 | +2.2 (-3.8 to 9.2) | no change |
| Conduct disorder | 69.0 | +0.6 (-6.4 to 8.0) | no change |
| Fire, heat, and hot substances | 65.9 | -49.2 (-58.0 to -40.6) | decrease |
| Interpersonal violence | 59.6 | -22.7 (-27.5 to -18.2) | decrease |
| Idiopathic developmental intellectual disability | 52.0 | -12.8 (-33.1 to 10.9) | no change |
| Atrial fibrillation and flutter | 51.7 | -1.9 (-8.8 to 4.9) | no change |
| Eating disorders | 51.7 | +32.8 (18.1 to 48.8) | increase |
| Drug use disorders | 47.8 | +5.4 (-7.0 to 18.1) | no change |
| Upper respiratory infections | 46.3 | -0.6 (-4.9 to 5.2) | no change |
| Urinary diseases and male infertility | 42.3 | +1.8 (-3.3 to 6.9) | no change |
| Psoriasis | 41.1 | +39.5 (24.5 to 53.8) | increase |
| Other cardiovascular and circulatory diseases | 29.0 | -11.6 (-24.4 to 3.3) | no change |
| Hemoglobinopathies and hemolytic anemias | 27.2 | -41.5 (-74.5 to 39.9) | no change |
| Acne vulgaris | 26.6 | +152.7 (124.7 to 186.4) | increase |
| Other neglected tropical diseases | 26.1 | -38.5 (-71.4 to 35.9) | no change |
| Other neurological disorders | 25.8 | +137.3 (3.6 to 576.9) | increase |

| Cause | Rate 2023 | Percentage change 1990 to 2023 (95% UI) | Direction |
|---|---|---|---|
| Attention-deficit/hyperactivity disorder | 24.8 | +0.8 (-3.7 to 5.3) | no change |
| Otitis media | 23.6 | -14.8 (-20.2 to -9.7) | decrease |
| Cirrhosis and other chronic liver diseases | 23.4 | -12.6 (-20.1 to -4.1) | decrease |
| Other unspecified infectious diseases | 22.5 | -35.5 (-70.2 to 40.3) | no change |
| Cysticercosis | 20.2 | -42.5 (-73.2 to 24.6) | no change |
| Breast cancer | 19.3 | +42.6 (15.9 to 69.9) | increase |
| Rheumatoid arthritis | 18.0 | +53.0 (34.4 to 74.1) | increase |
| Diarrheal diseases | 18.0 | -36.9 (-43.0 to -30.8) | decrease |
| Foreign body | 17.1 | -26.8 (-32.6 to -21.7) | decrease |
| Poisonings | 16.8 | -61.1 (-64.5 to -57.4) | decrease |
| Other skin and subcutaneous diseases | 15.8 | +21.7 (18.0 to 25.7) | increase |
| Environmental heat and cold exposure | 15.7 | -23.7 (-29.7 to -18.4) | decrease |
| Viral skin diseases | 15.5 | +2.8 (-4.9 to 12.0) | no change |
| Inguinal, femoral, and abdominal hernia | 15.3 | -34.8 (-44.6 to -24.4) | decrease |
| Parkinson's disease | 14.8 | +56.1 (35.4 to 84.5) | increase |
| Other transport injuries | 13.7 | -33.7 (-38.6 to -29.3) | decrease |
| Colon and rectum cancer | 13.4 | +106.6 (64.5 to 153.9) | increase |
| Other sense organ diseases | 12.9 | +4.9 (-5.5 to 13.8) | no change |
| Tuberculosis | 12.8 | -62.0 (-68.3 to -56.0) | decrease |
| Non-rheumatic valvular heart disease | 12.1 | +33.3 (17.8 to 51.3) | increase |
| Animal contact | 11.5 | -29.0 (-33.1 to -25.2) | decrease |
| Gout | 11.2 | +16.9 (1.5 to 32.6) | increase |
| Hypertensive heart disease | 11.1 | +19.2 (-1.1 to 43.2) | no change |
| Other chronic respiratory diseases | 10.5 | +34.1 (20.6 to 50.0) | increase |

| Cause | Rate 2023 | Percentage change 1990 to 2023 (95% UI) | Direction |
|---|---|---|---|
| Other digestive diseases | 9.4 | -9.0 (-15.5 to -1.8) | decrease |
| Cardiomyopathy and myocarditis | 8.8 | +6.3 (-10.9 to 22.1) | no change |
| Prostate cancer | 8.7 | +160.1 (83.7 to 270.5) | increase |
| Sexually transmitted infections excluding HIV | 8.4 | +0.5 (-6.6 to 9.4) | no change |
| Fungal skin diseases | 7.8 | -8.7 (-15.4 to -1.3) | decrease |
| Pancreatitis | 7.3 | +6.2 (-15.6 to 34.9) | no change |
| Self-harm | 7.1 | -31.0 (-38.2 to -25.0) | decrease |
| HIV/AIDS | 7.0 | +820.8 (577.9 to 1164.3) | increase |
| Tracheal, bronchus, and lung cancer | 6.7 | +13.3 (-7.0 to 35.0) | no change |
| Maternal disorders | 5.8 | -9.6 (-29.4 to 12.3) | no change |
| Acute hepatitis | 5.6 | -13.5 (-23.8 to -2.3) | decrease |
| Lower extremity peripheral arterial disease | 5.5 | -9.7 (-17.7 to -1.7) | decrease |
| Cervical cancer | 5.4 | -11.5 (-36.0 to 23.4) | no change |
| Multiple sclerosis | 4.8 | +0.5 (-13.0 to 18.2) | no change |
| Bladder cancer | 4.7 | +62.0 (24.5 to 109.1) | increase |
| Other neoplasms | 4.5 | +60.3 (32.8 to 94.1) | increase |
| Interstitial lung disease and pulmonary sarcoidosis | 4.3 | -22.0 (-35.5 to -6.8) | decrease |
| Iodine deficiency | 4.2 | -17.8 (-31.6 to -3.4) | decrease |
| Appendicitis | 4.1 | -0.6 (-25.1 to 37.6) | no change |
| Uterine cancer | 3.6 | +39.4 (1.0 to 95.1) | increase |
| Rheumatic heart disease | 3.3 | -37.3 (-47.5 to -25.9) | decrease |
| Scabies | 3.3 | -1.5 (-10.3 to 9.5) | no change |
| Leukemia | 2.9 | +21.2 (-11.9 to 74.1) | no change |
| Inflammatory bowel disease | 2.8 | +4.9 (-17.8 to 32.5) | no change |
| Non-Hodgkin lymphoma | 2.8 | +129.4 (22.4 to 344.0) | increase |
| Decubitus ulcer | 2.5 | -3.7 (-18.0 to 12.8) | no change |
| Larynx cancer | 2.4 | +21.9 (-7.5 to 59.4) | no change |

| Cause | Rate 2023 | Percentage change 1990 to 2023 (95% UI) | Direction |
|---|---|---|---|
| Kidney cancer | 2.4 | +116.9 (67.0 to 181.4) | increase |
| Stomach cancer | 2.4 | -30.8 (-44.4 to -12.6) | decrease |
| Ovarian cancer | 2.4 | +7.0 (-18.9 to 47.1) | no change |
| Lip and oral cavity cancer | 2.2 | +90.9 (48.7 to 147.3) | increase |
| Other malignant neoplasms | 2.2 | +22.6 (-11.1 to 68.9) | no change |
| Malignant skin melanoma | 2.1 | +165.3 (79.5 to 326.5) | increase |
| Adverse effects of medical treatment | 2.1 | +11.1 (7.1 to 15.9) | increase |
| Conflict and terrorism | 2.1 | -64.7 (-69.6 to -59.0) | decrease |
| Pancreatic cancer | 1.9 | +38.9 (16.5 to 67.5) | increase |
| Brain and central nervous system cancer | 1.9 | +19.2 (-4.7 to 53.0) | no change |
| Meningitis | 1.8 | -72.7 (-75.7 to -69.1) | decrease |
| Encephalitis | 1.8 | -43.8 (-48.6 to -37.5) | decrease |
| Alopecia areata | 1.8 | -0.5 (-17.4 to 18.9) | no change |
| Pruritus | 1.7 | +25.6 (16.2 to 38.1) | increase |
| Pertussis | 1.6 | +46.7 (24.0 to 69.5) | increase |
| Lower respiratory infections | 1.6 | -54.5 (-60.5 to -46.8) | decrease |
| Varicella and herpes zoster | 1.5 | -0.2 (-15.0 to 20.8) | no change |
| Drowning | 1.5 | -56.9 (-60.1 to -54.2) | decrease |
| Testicular cancer | 1.5 | +139.3 (39.1 to 362.8) | increase |
| Vitamin A deficiency | 1.4 | -89.1 (-96.6 to -68.7) | decrease |
| Paralytic ileus and intestinal obstruction | 1.4 | +25.8 (9.0 to 48.1) | increase |
| Vascular intestinal disorders | 1.4 | +58.9 (34.3 to 91.2) | increase |
| Electrocution | 1.4 | -63.3 (-67.7 to -59.7) | decrease |
| Bacterial skin diseases | 1.3 | -0.1 (-4.5 to 5.1) | no change |
| Malignant neoplasm of bone and articular cartilage | 1.1 | -51.8 (-66.9 to -26.9) | decrease |
| Thyroid cancer | 1.1 | +8.2 (-29.5 to 80.9) | no change |
| Other pharynx cancer | 1.1 | +182.1 (111.5 to 270.1) | increase |

| Cause | Rate 2023 | Percentage change 1990 to 2023 (95% UI) | Direction |
|---|---|---|---|
| Soft tissue and other extraosseous sarcomas | 0.9 | +31.8 (-2.8 to 78.0) | no change |
| Cystic echinococcosis | 0.8 | -8.3 (-20.0 to 0.3) | no change |
| Motor neuron disease | 0.7 | +14.6 (3.9 to 28.5) | increase |
| Multiple myeloma | 0.7 | +80.3 (13.5 to 226.2) | increase |
| Esophageal cancer | 0.7 | +56.0 (30.7 to 81.3) | increase |
| Liver cancer | 0.7 | +100.6 (66.0 to 144.3) | increase |
| Hodgkin lymphoma | 0.6 | -18.6 (-42.5 to 19.2) | no change |
| Nasopharynx cancer | 0.5 | +136.3 (66.7 to 256.5) | increase |
| Pneumoconiosis | 0.4 | +36.7 (-3.1 to 52.4) | no change |
| Gallbladder and biliary tract cancer | 0.3 | -30.9 (-39.5 to -22.3) | decrease |
| Non-melanoma skin cancer | 0.3 | -8.2 (-17.8 to 3.2) | no change |
| Protein-energy malnutrition | 0.2 | -88.5 (-96.5 to -68.0) | decrease |
| Exposure to forces of nature | 0.2 | +503.7 (353.8 to 731.3) | increase |
| Endocarditis | 0.2 | +169.0 (142.3 to 199.0) | increase |
| Pulmonary Arterial Hypertension | 0.2 | -13.7 (-17.8 to -9.0) | decrease |
| Eye cancer | 0.1 | -47.5 (-67.2 to -9.2) | decrease |
| Mesothelioma | 0.1 | +64.6 (43.2 to 88.4) | increase |
| Measles | 0.0 | -31.9 (-35.4 to -27.7) | decrease |
| Police conflict and executions | 0.0 | +420.4 (177.0 to 983.2) | increase |
| Neuroblastoma and other peripheral nervous cell tumors | 0.0 | -8.7 (-42.7 to 46.7) | no change |
| Acute glomerulonephritis | 0.0 | -8.0 (-14.6 to -1.8) | decrease |
| Other intestinal infectious diseases | 0.0 | +86.5 (64.8 to 108.6) | increase |
| Other nutritional deficiencies | 0.0 | -96.7 (-99.4 to -71.6) | decrease |
| Leishmaniasis | 0.0 | -97.8 (-99.6 to -89.9) | decrease |
| Typhoid and paratyphoid | 0.0 | -94.4 (-95.4 to -93.1) | decrease |
| Tetanus | 0.0 | -79.5 (-93.0 to -57.1) | decrease |

| Cause | Rate 2023 | Percentage change 1990 to 2023 (95% UI) | Direction |
|---|---|---|---|
| Invasive Non-typhoidal Salmonella (iNTS) | 0.0 | -1.0 (-11.6 to 8.2) | no change |
| Diphtheria | 0.0 | +502.3 (108.3 to 1594.0) | increase |
| Rabies | 0.0 | -99.8 (-99.9 to -99.6) | decrease |
| Yellow fever | 0.0 | +0.0 (0.0 to 0.0) | no change |
| Food-borne trematodiases | 0.0 | +0.0 (0.0 to 0.0) | no change |
| Intestinal nematode infections | 0.0 | +0.0 (0.0 to 0.0) | no change |
| Leprosy | 0.0 | +0.0 (0.0 to 0.0) | no change |
| Chagas disease | 0.0 | +0.0 (0.0 to 0.0) | no change |
| African trypanosomiasis | 0.0 | +0.0 (0.0 to 0.0) | no change |
| Malaria | 0.0 | +0.0 (0.0 to 0.0) | no change |
| Lymphatic filariasis | 0.0 | +0.0 (0.0 to 0.0) | no change |
| Onchocerciasis | 0.0 | +0.0 (0.0 to 0.0) | no change |
| Trachoma | 0.0 | +0.0 (0.0 to 0.0) | no change |
| Dengue | 0.0 | +0.0 (0.0 to 0.0) | no change |
| Schistosomiasis | 0.0 | +0.0 (0.0 to 0.0) | no change |
| Ebola | 0.0 | +0.0 (0.0 to 0.0) | no change |
| Guinea worm disease | 0.0 | +0.0 (0.0 to 0.0) | no change |
| Zika virus | 0.0 | +0.0 (0.0 to 0.0) | no change |

**Table S12. Age-standardised incidence by level 2 cause, Romania, 1990 and 2023.**

| Cause | Incidence 1990 (95% UI) | Incidence 2023 (95% UI) | Difference per 100 000 |
|---|---|---|---|
| All causes | 351,344 (331,589 to 372,561) | 418,970 (337,390 to 586,412) | +67,627 |
| Respiratory infections and tuberculosis | 142,150 (127,209 to 160,497) | 233,214 (151,738 to 400,643) | +91,065 |
| Other non-communicable diseases | 74,359 (67,818 to 80,840) | 68,035 (59,902 to 78,496) | -6,324 |
| Skin and subcutaneous diseases | 35,733 (34,575 to 36,954) | 39,123 (38,085 to 40,340) | +3,390 |
| Unintentional injuries | 22,365 (20,327 to 24,370) | 18,524 (16,589 to 20,323) | -3,841 |
| Neurological disorders | 11,699 (10,266 to 13,182) | 11,697 (10,284 to 13,175) | -2 |
| Enteric infections | 13,024 (10,747 to 15,607) | 9,717 (8,046 to 11,464) | -3,306 |
| Digestive diseases | 8,224 (7,634 to 8,793) | 7,837 (7,240 to 8,396) | -388 |
| Musculoskeletal disorders | 6,352 (5,781 to 7,031) | 6,136 (5,590 to 6,812) | -216 |
| HIV/AIDS and sexually transmitted infections | 5,616 (4,178 to 7,196) | 5,539 (4,044 to 7,056) | -77 |
| Other infectious diseases | 5,215 (4,873 to 5,549) | 4,361 (4,075 to 4,644) | -855 |
| Mental disorders | 3,082 (2,795 to 3,434) | 2,783 (2,521 to 3,177) | -299 |
| Nutritional deficiencies | 12,357 (5,816 to 21,724) | 2,579 (1,408 to 4,701) | -9,777 |
| Neoplasms | 2,490 (2,043 to 2,874) | 2,534 (2,092 to 2,920) | +45 |
| Maternal and neonatal disorders | 2,924 (2,564 to 3,328) | 2,504 (2,196 to 2,887) | -421 |
| Substance use disorders | 1,484 (1,271 to 1,715) | 1,212 (1,007 to 1,428) | -272 |
| Transport injuries | 1,381 (1,320 to 1,449) | 783 (740 to 827) | -599 |
| Chronic respiratory diseases | 768 (653 to 897) | 757 (633 to 894) | -11 |
| Cardiovascular diseases | 1,038 (982 to 1,101) | 614 (578 to 665) | -424 |
| Self-harm and interpersonal violence | 722 (674 to 778) | 589 (527 to 664) | -133 |
| Diabetes and kidney diseases | 350 (333 to 369) | 423 (402 to 442) | +73 |
| Sense organ diseases | 7 (3 to 11) | 7 (4 to 12) | +1 |
| Neglected tropical diseases and malaria | 4 (3 to 5) | 3 (2 to 3) | -1 |

**Table S13. Age-standardised prevalence by level 2 cause, Romania, 1990 and 2023.**

| Cause | Prevalence 1990 (95% UI) | Prevalence 2023 (95% UI) | 2023 as % of 1990 |
|---|---|---|---|
| All causes | 97,553 (96,980 to 98,061) | 95,134 (94,366 to 95,872) | 97.5 |
| Other non-communicable diseases | 76,218 (73,273 to 79,802) | 71,559 (68,113 to 75,286) | 93.9 |
| Neurological disorders | 39,307 (35,796 to 43,393) | 39,278 (35,592 to 43,405) | 99.9 |
| Unintentional injuries | 36,097 (34,084 to 38,141) | 31,114 (29,248 to 33,251) | 86.2 |
| Digestive diseases | 28,611 (27,258 to 30,127) | 26,319 (25,074 to 27,814) | 92.0 |
| Sense organ diseases | 21,208 (20,158 to 22,432) | 21,210 (20,258 to 22,355) | 100.0 |
| Musculoskeletal disorders | 20,480 (18,885 to 21,915) | 20,583 (19,183 to 22,030) | 100.5 |
| Respiratory infections and tuberculosis | 35,052 (31,398 to 38,634) | 19,124 (15,399 to 24,779) | 54.6 |
| Nutritional deficiencies | 30,798 (22,189 to 42,209) | 16,158 (12,113 to 22,152) | 52.5 |
| Mental disorders | 10,954 (9,754 to 12,353) | 12,599 (10,986 to 14,737) | 115.0 |
| Skin and subcutaneous diseases | 10,043 (9,669 to 10,492) | 11,227 (10,879 to 11,607) | 111.8 |
| HIV/AIDS and sexually transmitted infections | 8,670 (7,650 to 9,766) | 8,752 (7,709 to 9,863) | 100.9 |
| Diabetes and kidney diseases | 7,153 (6,823 to 7,463) | 8,050 (7,723 to 8,359) | 112.5 |
| Cardiovascular diseases | 7,649 (7,184 to 8,531) | 6,308 (5,958 to 6,827) | 82.5 |
| Chronic respiratory diseases | 6,373 (5,606 to 7,287) | 6,280 (5,498 to 7,129) | 98.5 |
| Neoplasms | 4,459 (3,803 to 5,157) | 4,793 (4,200 to 5,511) | 107.5 |
| Self-harm and interpersonal violence | 3,088 (2,832 to 3,330) | 2,824 (2,589 to 3,059) | 91.5 |
| Substance use disorders | 2,940 (2,623 to 3,392) | 2,322 (2,007 to 2,726) | 79.0 |
| Maternal and neonatal disorders | 1,955 (1,629 to 2,339) | 2,284 (2,036 to 2,689) | 116.9 |
| Transport injuries | 3,587 (3,383 to 3,790) | 2,030 (1,914 to 2,169) | 56.6 |
| Other infectious diseases | 1,963 (1,462 to 2,733) | 1,311 (1,034 to 1,707) | 66.8 |
| Neglected tropical diseases and malaria | 1,869 (1,319 to 2,730) | 1,260 (933 to 1,703) | 67.4 |
| Enteric infections | 246 (209 to 290) | 157 (133 to 189) | 64.0 |

***Table S14*. Age-specific rate of years lived with disability from all causes, Romania, 1990 and 2023.**

| Age group | Both 1990 | Both 2023 | Male 2023 | Female 2023 | 2023 as % of 1990 |
|---|---|---|---|---|---|
| <5 years | 3,742.5 | 3,213.8 | 3,377.0 | 3,040.9 | 85.9 |
| 5-9 years | 4,255.9 | 4,099.2 | 3,980.1 | 4,225.0 | 96.3 |
| 10-14 years | 5,864.2 | 5,896.4 | 5,309.2 | 6,518.5 | 100.5 |
| 15-19 years | 7,368.2 | 7,183.3 | 6,295.5 | 8,122.4 | 97.5 |
| 20-24 years | 8,235.5 | 7,947.5 | 6,915.5 | 9,035.5 | 96.5 |
| 25-29 years | 9,222.9 | 8,763.7 | 7,683.9 | 9,894.1 | 95.0 |
| 30-34 years | 10,399.6 | 9,739.6 | 8,695.2 | 10,845.5 | 93.7 |
| 35-39 years | 11,722.8 | 10,870.5 | 9,920.5 | 11,886.1 | 92.7 |
| 40-44 years | 12,994.5 | 12,131.3 | 11,207.7 | 13,113.8 | 93.4 |
| 45-49 years | 14,572.7 | 13,661.7 | 12,738.7 | 14,627.2 | 93.7 |
| 50-54 years | 16,592.8 | 15,636.8 | 14,855.8 | 16,424.5 | 94.2 |
| 55-59 years | 18,651.5 | 17,827.2 | 17,473.5 | 18,169.2 | 95.6 |
| 60-64 years | 20,769.2 | 20,428.1 | 20,515.0 | 20,352.6 | 98.4 |
| 65-69 years | 23,168.7 | 22,933.8 | 23,642.6 | 22,382.1 | 99.0 |
| 70-74 years | 25,866.3 | 25,562.9 | 26,698.6 | 24,757.5 | 98.8 |
| 75-79 years | 28,148.9 | 28,013.1 | 29,376.4 | 27,149.0 | 99.5 |
| 80-84 years | 30,405.2 | 30,444.0 | 31,849.2 | 29,698.0 | 100.1 |
| 85-89 years | 32,564.9 | 32,569.4 | 33,754.1 | 32,034.1 | 100.0 |
| 90-94 years | 34,615.1 | 34,425.2 | 35,457.8 | 33,962.9 | 99.5 |
| 95+ years | 37,151.6 | 36,935.6 | 38,110.2 | 36,471.4 | 99.4 |

*Table S15*. **Healthy life expectancy and life expectancy at birth, Romania, both sexes, 1990 to 2023.** *Years in poor health is the point-estimate difference between life expectancy and HALE; no uncertainty interval for this correlated difference was calculated.*

| Year | HALE (95% UI) | Life expectancy (95% UI) | Years in poor health |
|---|---|---|---|
| 1990 | 60.88 (58.29 to 63.11) | 70.08 (69.98 to 70.20) | 9.20 |
| 1991 | 60.78 (58.22 to 62.98) | 69.90 (69.80 to 70.00) | 9.12 |
| 1992 | 60.63 (58.12 to 62.79) | 69.66 (69.56 to 69.76) | 9.03 |
| 1993 | 60.61 (58.07 to 62.76) | 69.61 (69.51 to 69.71) | 9.00 |
| 1994 | 60.59 (58.07 to 62.78) | 69.58 (69.46 to 69.68) | 8.98 |
| 1995 | 60.41 (57.85 to 62.63) | 69.31 (69.19 to 69.42) | 8.90 |
| 1996 | 60.26 (57.77 to 62.44) | 69.07 (68.96 to 69.18) | 8.81 |
| 1997 | 60.51 (58.02 to 62.65) | 69.36 (69.26 to 69.46) | 8.85 |
| 1998 | 61.01 (58.49 to 63.16) | 69.98 (69.87 to 70.08) | 8.97 |
| 1999 | 61.53 (59.04 to 63.71) | 70.59 (70.49 to 70.70) | 9.06 |
| 2000 | 61.94 (59.40 to 64.11) | 71.09 (70.99 to 71.20) | 9.15 |
| 2001 | 62.07 (59.57 to 64.26) | 71.20 (71.10 to 71.30) | 9.14 |
| 2002 | 62.12 (59.64 to 64.31) | 71.20 (71.09 to 71.29) | 9.07 |
| 2003 | 62.41 (59.90 to 64.62) | 71.51 (71.41 to 71.61) | 9.10 |
| 2004 | 62.87 (60.39 to 65.13) | 72.06 (71.95 to 72.16) | 9.18 |
| 2005 | 63.15 (60.65 to 65.36) | 72.37 (72.27 to 72.46) | 9.22 |
| 2006 | 63.51 (60.96 to 65.79) | 72.80 (72.70 to 72.89) | 9.28 |
| 2007 | 63.86 (61.22 to 66.15) | 73.19 (73.09 to 73.29) | 9.33 |
| 2008 | 64.07 (61.50 to 66.33) | 73.42 (73.32 to 73.52) | 9.35 |
| 2009 | 64.22 (61.65 to 66.41) | 73.56 (73.45 to 73.66) | 9.34 |
| 2010 | 64.49 (61.80 to 66.74) | 73.87 (73.78 to 73.97) | 9.38 |
| 2011 | 64.90 (62.23 to 67.23) | 74.41 (74.31 to 74.50) | 9.50 |
| 2012 | 65.17 (62.53 to 67.44) | 74.76 (74.67 to 74.86) | 9.59 |
| 2013 | 65.44 (62.79 to 67.72) | 75.12 (75.03 to 75.21) | 9.68 |
| 2014 | 65.46 (62.82 to 67.79) | 75.16 (75.06 to 75.25) | 9.69 |
| 2015 | 65.47 (62.81 to 67.77) | 75.16 (75.06 to 75.25) | 9.69 |
| 2016 | 65.63 (62.99 to 67.95) | 75.33 (75.24 to 75.42) | 9.70 |
| 2017 | 65.69 (63.01 to 68.00) | 75.36 (75.27 to 75.45) | 9.67 |
| 2018 | 65.78 (63.13 to 68.04) | 75.42 (75.32 to 75.51) | 9.64 |
| 2019 | 66.02 (63.36 to 68.32) | 75.70 (75.61 to 75.78) | 9.67 |
| 2020 | 64.70 (61.94 to 66.96) | 74.32 (74.28 to 74.36) | 9.62 |
| 2021 | 63.53 (60.75 to 65.74) | 73.00 (72.95 to 73.04) | 9.46 |
| 2022 | 65.25 (62.36 to 67.60) | 75.27 (75.14 to 75.39) | 10.02 |
| 2023 | 66.05 (63.15 to 68.41) | 76.26 (76.13 to 76.39) | 10.21 |

***Table S16*. Healthy life expectancy and life expectancy at birth and at 65 to 69 years by sex, Romania, 1990 and 2023.**

| Measure and age | Sex | 1990 (95% UI) | 2023 (95% UI) |
|---|---|---|---|
| HALE at birth | Both sexes | 60.88 (58.29 to 63.11) | 66.05 (63.15 to 68.41) |
| HALE at birth | Male | 58.53 (56.08 to 60.57) | 63.54 (61.09 to 65.69) |
| HALE at birth | Female | 63.30 (60.45 to 65.69) | 68.62 (65.25 to 71.35) |
| Life expectancy at birth | Both sexes | 70.08 (69.98 to 70.20) | 76.26 (76.13 to 76.39) |
| Life expectancy at birth | Male | 66.94 (66.78 to 67.11) | 72.49 (72.32 to 72.69) |
| Life expectancy at birth | Female | 73.34 (73.21 to 73.47) | 80.14 (79.98 to 80.29) |
| HALE at 65 to 69 years | Both sexes | 10.52 (9.54 to 11.39) | 12.56 (11.40 to 13.61) |
| HALE at 65 to 69 years | Male | 9.53 (8.61 to 10.40) | 10.88 (9.84 to 11.84) |
| HALE at 65 to 69 years | Female | 11.31 (10.29 to 12.21) | 13.92 (12.58 to 15.11) |
| Life expectancy at 65 to 69 years | Both sexes | 14.32 (14.25 to 14.40) | 17.22 (17.13 to 17.30) |
| Life expectancy at 65 to 69 years | Male | 13.15 (13.05 to 13.26) | 15.07 (14.93 to 15.21) |
| Life expectancy at 65 to 69 years | Female | 15.25 (15.15 to 15.35) | 18.97 (18.85 to 19.08) |

*Table S17*. **Attributable fraction and attributable rate of years lived with disability, all risk factors, Romania, 1990 to 2023.**

| Year | PAF (%, 95% UI) | Attributable rate per 100 000 (95% UI) |
|---|---|---|
| 1990 | 31.19 (26.54 to 35.44) | 3,493.6 (2,530.4 to 4,675.3) |
| 1991 | 31.19 (26.60 to 35.58) | 3,485.2 (2,523.3 to 4,694.2) |
| 1992 | 31.13 (26.62 to 35.49) | 3,471.1 (2,509.1 to 4,704.9) |
| 1993 | 31.13 (26.65 to 35.55) | 3,463.4 (2,503.5 to 4,731.7) |
| 1994 | 31.06 (26.56 to 35.44) | 3,448.7 (2,498.0 to 4,715.5) |
| 1995 | 31.00 (26.48 to 35.29) | 3,434.4 (2,482.4 to 4,690.3) |
| 1996 | 31.02 (26.50 to 35.27) | 3,429.1 (2,483.3 to 4,677.0) |
| 1997 | 31.04 (26.54 to 35.18) | 3,420.2 (2,472.1 to 4,659.9) |
| 1998 | 31.13 (26.69 to 35.18) | 3,418.2 (2,465.5 to 4,694.7) |
| 1999 | 31.25 (26.99 to 35.28) | 3,413.5 (2,463.4 to 4,716.1) |
| 2000 | 31.20 (27.16 to 35.28) | 3,397.4 (2,444.6 to 4,670.8) |
| 2001 | 30.99 (27.01 to 35.00) | 3,363.7 (2,410.6 to 4,616.9) |
| 2002 | 30.74 (26.90 to 34.73) | 3,320.4 (2,376.9 to 4,506.5) |
| 2003 | 30.49 (26.70 to 34.64) | 3,278.9 (2,328.1 to 4,464.5) |
| 2004 | 30.26 (26.53 to 34.65) | 3,238.1 (2,299.9 to 4,413.7) |
| 2005 | 30.06 (26.28 to 34.58) | 3,209.1 (2,277.1 to 4,382.1) |
| 2006 | 29.91 (26.24 to 34.21) | 3,180.5 (2,268.1 to 4,325.5) |
| 2007 | 29.72 (26.12 to 33.98) | 3,142.5 (2,263.3 to 4,254.0) |
| 2008 | 29.47 (25.85 to 33.56) | 3,104.6 (2,248.1 to 4,174.6) |
| 2009 | 29.32 (25.79 to 33.42) | 3,075.6 (2,232.1 to 4,110.3) |
| 2010 | 29.25 (25.67 to 33.14) | 3,061.0 (2,231.5 to 4,078.7) |
| 2011 | 29.29 (25.70 to 33.21) | 3,063.2 (2,235.2 to 4,078.8) |
| 2012 | 29.21 (25.64 to 33.09) | 3,057.2 (2,237.1 to 4,083.3) |
| 2013 | 29.23 (25.71 to 33.05) | 3,055.1 (2,238.9 to 4,088.4) |
| 2014 | 29.11 (25.52 to 33.02) | 3,045.0 (2,235.7 to 4,075.5) |
| 2015 | 29.08 (25.47 to 33.05) | 3,039.9 (2,223.7 to 4,074.3) |
| 2016 | 29.11 (25.53 to 33.11) | 3,033.6 (2,213.0 to 4,053.8) |
| 2017 | 29.14 (25.57 to 33.09) | 3,024.8 (2,190.5 to 4,042.1) |
| 2018 | 29.19 (25.61 to 33.20) | 3,012.9 (2,152.5 to 4,036.8) |
| 2019 | 29.23 (25.56 to 33.35) | 3,002.7 (2,129.4 to 4,043.7) |
| 2020 | 28.21 (24.57 to 32.30) | 3,012.1 (2,118.9 to 4,074.3) |
| 2021 | 27.53 (23.71 to 31.58) | 3,009.6 (2,121.4 to 4,078.8) |
| 2022 | 27.74 (23.65 to 32.00) | 3,010.7 (2,108.6 to 4,073.8) |
| 2023 | 28.26 (24.21 to 32.52) | 3,034.6 (2,122.4 to 4,102.3) |

*Table S18*. **Attributable fraction of years lived with disability by risk factor, Romania, 1990 and 2023.**

| Risk factor | *Rank 2023 within level* | PAF 1990 (%, 95% UI) | PAF 2023 (%, 95% UI) | *Level* |
|---|---|---|---|---|
| All risk factors | - | 31.19 (26.54 to 35.44) | 28.26 (24.21 to 32.52) | - |
| Behavioral risks | *1* | 19.12 (16.00 to 22.48) | 16.58 (14.11 to 19.13) | *1* |
| Metabolic risks | *2* | 9.76 (7.86 to 11.81) | 10.87 (8.42 to 13.19) | *1* |
| Environmental/occupational risks | *3* | 8.72 (3.24 to 13.75) | 6.33 (2.05 to 10.37) | *1* |
| High body-mass index | *1* | 3.74 (1.17 to 6.42) | 5.29 (1.90 to 8.52) | *2* |
| Tobacco | *2* | 5.53 (3.88 to 7.36) | 5.01 (3.62 to 6.65) | *2* |
| Smoking | *1* | 5.02 (3.35 to 6.96) | 4.57 (3.14 to 6.24) | *3* |
| Occupational risks | *3* | 5.31 (-0.17 to 10.39) | 4.35 (0.08 to 8.14) | *2* |
| High fasting plasma glucose | *4* | 2.53 (2.08 to 3.10) | 3.91 (3.28 to 4.64) | *2* |
| Child and maternal malnutrition | *5* | 5.56 (3.48 to 8.83) | 3.74 (2.56 to 5.61) | *2* |
| High alcohol use | *6* | 3.71 (3.01 to 4.45) | 2.99 (2.46 to 3.67) | *2* |
| Iron deficiency | *2* | 4.23 (2.13 to 7.69) | 2.65 (1.50 to 4.60) | *3* |
| High systolic blood pressure | *7* | 2.94 (2.31 to 3.54) | 2.23 (1.68 to 2.77) | *2* |
| Occupational ergonomic factors | *3* | 2.56 (-3.04 to 7.62) | 2.19 (-1.92 to 5.96) | *3* |
| Dietary risks | *8* | 2.11 (1.03 to 2.88) | 2.13 (1.14 to 2.93) | *2* |
| Sexual violence against children and bullying | *9* | 1.76 (0.71 to 3.13) | 1.78 (0.88 to 2.92) | *2* |
| Low bone mineral density | *10* | 1.64 (1.36 to 1.91) | 1.52 (1.21 to 1.82) | *2* |
| Air pollution | *11* | 2.79 (1.96 to 3.49) | 1.45 (1.04 to 1.99) | *2* |
| Particulate matter pollution | *4* | 2.75 (1.92 to 3.45) | 1.43 (1.03 to 1.93) | *3* |
| Sexual violence against children | *5* | 1.37 (0.44 to 2.74) | 1.37 (0.54 to 2.41) | *3* |
| Occupational injuries | *6* | 1.73 (1.45 to 2.08) | 1.24 (0.87 to 1.68) | *3* |
| Kidney dysfunction | *12* | 0.90 (0.73 to 1.05) | 1.13 (0.93 to 1.31) | *2* |
| Low birth weight and short gestation | *7* | 1.13 (0.88 to 1.42) | 1.05 (0.83 to 1.27) | *3* |
| High LDL cholesterol | *13* | 1.37 (0.78 to 2.01) | 0.88 (0.49 to 1.29) | *2* |
| Intimate partner violence | *14* | 0.63 (0.17 to 1.37) | 0.80 (0.17 to 1.66) | *2* |
| Occupational noise | *8* | 0.73 (0.59 to 0.89) | 0.68 (0.55 to 0.84) | *3* |
| Secondhand smoke | *9* | 0.66 (0.51 to 0.84) | 0.56 (0.44 to 0.70) | *3* |

| Risk factor | *Rank 2023 within level* | PAF 1990 (%, 95% UI) | PAF 2023 (%, 95% UI) | *Level* |
|---|---|---|---|---|
| Diet high in sodium | *10* | 0.79 (0.25 to 1.41) | 0.52 (0.13 to 1.01) | *3* |
| Diet high in processed meat | *11* | 0.25 (0.10 to 0.42) | 0.50 (0.25 to 0.82) | *3* |
| Drug use | *15* | 0.42 (0.32 to 0.53) | 0.49 (0.37 to 0.62) | *2* |
| Other environmental risks | *16* | 0.56 (0.38 to 0.84) | 0.49 (0.33 to 0.74) | *2* |
| Lead exposure | *12* | 0.56 (0.38 to 0.84) | 0.49 (0.33 to 0.74) | *3* |
| Bullying victimization | *13* | 0.42 (0.18 to 0.79) | 0.43 (0.20 to 0.77) | *3* |
| Diet high in sugar-sweetened beverages | *14* | 0.11 (0.04 to 0.22) | 0.33 (0.14 to 0.61) | *3* |
| Low physical activity | *17* | 0.26 (0.11 to 0.41) | 0.32 (0.13 to 0.52) | *2* |
| Diet high in red meat | *15* | 0.17 (0.00 to 0.35) | 0.30 (0.00 to 0.64) | *3* |
| Diet low in fruits | *16* | 0.29 (0.11 to 0.48) | 0.22 (0.09 to 0.42) | *3* |
| Diet low in whole grains | *17* | 0.21 (0.03 to 0.40) | 0.20 (0.04 to 0.39) | *3* |
| Unsafe sex | *18* | 0.13 (0.10 to 0.18) | 0.17 (0.13 to 0.22) | *2* |
| Diet low in nuts and seeds | *18* | 0.31 (0.10 to 0.51) | 0.17 (0.05 to 0.29) | *3* |
| Occupational asthmagens | *19* | 0.16 (0.12 to 0.22) | 0.13 (0.09 to 0.17) | *3* |
| Diet low in legumes | *20* | 0.17 (-0.15 to 0.45) | 0.11 (-0.10 to 0.30) | *3* |
| Occupational particulate matter, gases, and fumes | *21* | 0.12 (0.04 to 0.21) | 0.10 (0.04 to 0.18) | *3* |
| Diet low in seafood omega-3 fatty acids | *22* | 0.19 (0.05 to 0.36) | 0.10 (0.02 to 0.19) | *3* |
| Unsafe water, sanitation, and handwashing | *19* | 0.21 (0.15 to 0.26) | 0.09 (0.06 to 0.15) | *2* |
| Diet low in omega-6 polyunsaturated fatty acids | *23* | 0.14 (-0.41 to 0.54) | 0.09 (-0.22 to 0.34) | *3* |
| Unsafe water source | *24* | 0.15 (0.06 to 0.24) | 0.07 (0.02 to 0.13) | *3* |
| Diet high in trans fatty acids | *25* | 0.11 (0.00 to 0.21) | 0.06 (0.00 to 0.11) | *3* |
| Unsafe sanitation | *26* | 0.12 (0.09 to 0.15) | 0.04 (0.03 to 0.06) | *3* |
| Diet low in vegetables | *27* | 0.08 (0.02 to 0.16) | 0.04 (0.00 to 0.13) | *3* |
| Diet low in fiber | *28* | 0.04 (0.01 to 0.10) | 0.02 (0.00 to 0.07) | *3* |
| Nitrogen dioxide pollution | *29* | 0.04 (-0.03 to 0.16) | 0.02 (-0.02 to 0.09) | *3* |
| Suboptimal breastfeeding | *30* | 0.04 (0.03 to 0.05) | 0.02 (0.01 to 0.02) | *3* |
| Vitamin A deficiency | *31* | 0.12 (0.05 to 0.23) | 0.01 (0.01 to 0.03) | *3* |
| Child growth failure | *32* | 0.05 (-0.01 to 0.11) | 0.01 (-0.01 to 0.03) | *3* |

| Risk factor | *Rank 2023 within level* | PAF 1990 (%, 95% UI) | PAF 2023 (%, 95% UI) | *Level* |
|---|---|---|---|---|
| Occupational carcinogens | *33* | 0.01 (0.01 to 0.01) | 0.01 (0.01 to 0.02) | *3* |
| Diet low in milk | *34* | 0.01 (0.00 to 0.01) | 0.01 (0.00 to 0.01) | *3* |
| No access to handwashing facility | *35* | 0.02 (0.00 to 0.04) | 0.00 (0.00 to 0.01) | *3* |
| Diet low in calcium | *36* | 0.00 (0.00 to 0.00) | 0.00 (0.00 to 0.00) | *3* |
| Residential radon | *37* | 0.00 (-0.00 to 0.01) | 0.00 (-0.00 to 0.01) | *3* |
| Chewing tobacco | *38* | 0.00 (0.00 to 0.01) | 0.00 (0.00 to 0.01) | *3* |
| Zinc deficiency | *39* | 0.00 (-0.00 to 0.01) | 0.00 (-0.00 to 0.00) | *3* |

*Table S19*. **Percentage change in the attributable fraction of years lived with disability by risk factor, Romania, 1990 to 2023.** *Relative changes become unstable when the 1990 fraction lies near zero and are read together with the absolute percentage-point changes.*

| Risk factor | PAF 2023 (%) | Percentage change 1990 to 2023 (95% UI) | Direction |
|---|---|---|---|
| All risk factors | 28.26 | -9.4 (-16.8 to -0.7) | decrease |
| Behavioral risks | 16.58 | -13.3 (-27.1 to 2.1) | no change |
| Metabolic risks | 10.87 | +11.4 (-0.7 to 24.2) | no change |
| Environmental/occupational risks | 6.33 | -27.4 (-39.7 to -17.8) | decrease |
| High body-mass index | 5.29 | +41.6 (2.0 to 91.4) | increase |
| Tobacco | 5.01 | -9.3 (-29.9 to 13.3) | no change |
| Smoking | 4.57 | -8.9 (-32.0 to 17.4) | no change |
| Occupational risks | 4.35 | -18.0 (-56.2 to 8.7) | no change |
| High fasting plasma glucose | 3.91 | +54.5 (25.9 to 83.9) | increase |
| Child and maternal malnutrition | 3.74 | -32.8 (-62.0 to 21.5) | no change |
| High alcohol use | 2.99 | -19.3 (-29.7 to -8.8) | decrease |
| Iron deficiency | 2.65 | -37.3 (-72.3 to 39.1) | no change |
| High systolic blood pressure | 2.23 | -24.1 (-40.8 to -4.4) | decrease |
| Occupational ergonomic factors | 2.19 | -14.4 (-83.4 to 30.1) | no change |
| Dietary risks | 2.13 | +0.8 (-19.1 to 24.1) | no change |
| Sexual violence against children and bullying | 1.78 | +1.3 (-31.5 to 59.4) | no change |
| Low bone mineral density | 1.52 | -7.3 (-14.8 to -0.5) | decrease |
| Air pollution | 1.45 | -47.9 (-60.4 to -33.0) | decrease |
| Particulate matter pollution | 1.43 | -47.9 (-60.3 to -32.7) | decrease |
| Sexual violence against children | 1.37 | +0.2 (-36.2 to 89.7) | no change |
| Occupational injuries | 1.24 | -28.5 (-47.5 to -2.5) | decrease |
| Kidney dysfunction | 1.13 | +24.6 (8.5 to 42.7) | increase |
| Low birth weight and short gestation | 1.05 | -7.4 (-24.1 to 13.5) | no change |
| High LDL cholesterol | 0.88 | -36.0 (-47.4 to -23.4) | decrease |
| Intimate partner violence | 0.80 | +26.2 (-31.1 to 114.7) | no change |
| Occupational noise | 0.68 | -7.2 (-14.4 to 0.8) | no change |
| Secondhand smoke | 0.56 | -15.1 (-27.9 to 1.3) | no change |

| Risk factor | PAF 2023 (%) | Percentage change 1990 to 2023 (95% UI) | Direction |
|---|---|---|---|
| Diet high in sodium | 0.52 | -34.2 (-70.1 to -1.8) | decrease |
| Diet high in processed meat | 0.50 | +101.5 (17.7 to 261.2) | increase |
| Drug use | 0.49 | +17.1 (2.8 to 34.7) | increase |
| Other environmental risks | 0.49 | -13.3 (-24.0 to -0.9) | decrease |
| Lead exposure | 0.49 | -13.5 (-24.1 to -1.0) | decrease |
| Bullying victimization | 0.43 | +3.7 (-30.3 to 55.1) | no change |
| Diet high in sugar-sweetened beverages | 0.33 | +200.0 (28.3 to 804.0) | increase |
| Low physical activity | 0.32 | +23.3 (-5.8 to 61.0) | no change |
| Diet high in red meat | 0.30 | +75.5 (-110.4 to 365.7) | no change |
| Diet low in fruits | 0.22 | -25.4 (-63.7 to 44.4) | no change |
| Diet low in whole grains | 0.20 | -2.5 (-51.4 to 103.3) | no change |
| Unsafe sex | 0.17 | +29.4 (12.5 to 56.8) | increase |
| Diet low in nuts and seeds | 0.17 | -44.5 (-55.5 to -34.0) | decrease |
| Occupational asthmagens | 0.13 | -18.3 (-28.5 to -5.1) | decrease |
| Diet low in legumes | 0.11 | -33.1 (-48.9 to -17.3) | decrease |
| Occupational particulate matter, gases, and fumes | 0.10 | -13.3 (-25.3 to -0.2) | decrease |
| Diet low in seafood omega-3 fatty acids | 0.10 | -49.5 (-71.1 to -22.8) | decrease |
| Unsafe water, sanitation, and handwashing | 0.09 | -54.4 (-66.6 to -42.0) | decrease |
| Diet low in omega-6 polyunsaturated fatty acids | 0.09 | -37.5 (-52.4 to -25.6) | decrease |
| Unsafe water source | 0.07 | -57.5 (-75.7 to -42.0) | decrease |
| Diet high in trans fatty acids | 0.06 | -45.4 (-64.8 to -16.4) | decrease |
| Unsafe sanitation | 0.04 | -63.2 (-73.0 to -52.7) | decrease |
| Diet low in vegetables | 0.04 | -50.5 (-97.7 to 73.1) | no change |
| Diet low in fiber | 0.02 | -46.0 (-92.1 to 119.2) | no change |
| Nitrogen dioxide pollution | 0.02 | -48.2 (-68.2 to -24.5) | decrease |
| Suboptimal breastfeeding | 0.02 | -58.4 (-63.9 to -52.2) | decrease |
| Vitamin A deficiency | 0.01 | -88.6 (-96.4 to -67.6) | decrease |

| Risk factor | PAF 2023 (%) | Percentage change 1990 to 2023 (95% UI) | Direction |
|---|---|---|---|
| Child growth failure | 0.01 | -74.3 (-120.5 to -60.8) | decrease |
| Occupational carcinogens | 0.01 | +24.9 (3.6 to 45.3) | increase |
| Diet low in milk | 0.01 | +18.8 (-39.0 to 134.7) | no change |
| No access to handwashing facility | 0.00 | -78.5 (-89.5 to -53.1) | decrease |
| Diet low in calcium | 0.00 | +7.2 (-22.0 to 41.2) | no change |
| Residential radon | 0.00 | +17.4 (-2.5 to 42.5) | no change |
| Chewing tobacco | 0.00 | +1.1 (-63.1 to 215.8) | no change |
| Zinc deficiency | 0.00 | -64.4 (-79.4 to -52.3) | decrease |

*Table S20*. **Attributable fraction of years lived with disability, all risk factors, *eighteen locations*, 1990 and 2023.**

| Location | PAF 1990 (%, 95% UI) | PAF 2023 (%, 95% UI) | Difference (pp) |
|---|---|---|---|
| Poland | 33.24 (29.76 to 36.46) | 30.74 (26.99 to 34.42) | -2.50 |
| Global | 32.36 (29.61 to 35.06) | 30.28 (27.27 to 33.25) | -2.09 |
| Bulgaria | 30.78 (27.35 to 33.74) | 30.08 (26.05 to 33.74) | -0.70 |
| North Macedonia | 30.90 (27.49 to 34.22) | 29.92 (26.35 to 33.50) | -0.98 |
| Czechia | 33.49 (29.99 to 36.86) | 29.63 (25.54 to 33.13) | -3.86 |
| Montenegro | 29.47 (26.24 to 32.39) | 29.46 (25.52 to 32.93) | -0.01 |
| Central Europe | 31.99 (28.52 to 35.23) | 29.46 (25.71 to 33.12) | -2.53 |
| Bosnia and Herzegovina | 31.95 (28.86 to 35.06) | 29.32 (25.88 to 33.22) | -2.63 |
| Central Europe, Eastern Europe, and Central Asia | 31.37 (28.37 to 34.47) | 29.30 (25.71 to 32.71) | -2.07 |
| Hungary | 32.82 (29.35 to 35.96) | 29.20 (25.22 to 32.74) | -3.63 |
| Croatia | 29.10 (25.99 to 32.10) | 28.91 (25.30 to 32.36) | -0.18 |
| Slovenia | 28.88 (25.49 to 32.06) | 28.66 (25.29 to 31.95) | -0.21 |
| Serbia | 30.46 (27.05 to 34.01) | 28.33 (24.47 to 32.16) | -2.13 |
| Romania | 31.19 (26.54 to 35.44) | 28.26 (24.21 to 32.52) | -2.93 |
| Eastern Europe | 30.13 (27.13 to 33.12) | 28.02 (24.70 to 31.21) | -2.10 |
| Slovakia | 29.94 (26.13 to 33.36) | 27.44 (23.62 to 30.89) | -2.50 |
| Albania | 31.26 (27.18 to 35.18) | 26.90 (22.45 to 30.91) | -4.36 |
| Western Europe | 28.90 (25.81 to 31.70) | 26.42 (22.82 to 29.51) | -2.48 |

*Table S21*. **Attributable fraction of years lived with disability by risk factor, Romania against regional aggregates, 2023.**

| Risk factor | Romania | Central Europe | Eastern Europe | Western Europe | Global | Romania minus Central Europe (pp) |
|---|---|---|---|---|---|---|
| All risk factors | 28.26 | 29.46 | 28.02 | 26.42 | 30.28 | -1.20 |
| Behavioral risks | 16.58 | 17.25 | 16.74 | 15.96 | 17.60 | -0.66 |
| Metabolic risks | 10.87 | 12.56 | 11.18 | 10.45 | 11.00 | -1.69 |
| Environmental/occupational risks | 6.33 | 6.18 | 5.50 | 4.28 | 7.02 | +0.15 |
| High body-mass index | 5.29 | 6.59 | 5.70 | 5.42 | 4.75 | -1.30 |
| Tobacco | 5.01 | 5.51 | 3.98 | 3.65 | 2.89 | -0.49 |
| Occupational risks | 4.35 | 4.00 | 3.60 | 3.23 | 3.59 | +0.35 |
| High fasting plasma glucose | 3.91 | 5.29 | 3.63 | 3.68 | 5.21 | -1.38 |
| Child and maternal malnutrition | 3.74 | 3.17 | 2.60 | 2.15 | 6.38 | +0.56 |
| High alcohol use | 2.99 | 3.19 | 3.28 | 2.54 | 1.59 | -0.20 |
| High systolic blood pressure | 2.23 | 2.10 | 2.55 | 1.50 | 1.81 | +0.13 |
| Dietary risks | 2.13 | 2.50 | 2.29 | 1.88 | 2.06 | -0.37 |
| Sexual violence against children and bullying | 1.78 | 1.98 | 2.31 | 3.56 | 2.59 | -0.20 |
| Low bone mineral density | 1.52 | 1.60 | 1.32 | 1.77 | 1.39 | -0.09 |
| Air pollution | 1.45 | 1.61 | 1.04 | 0.71 | 2.33 | -0.16 |
| Kidney dysfunction | 1.13 | 0.86 | 1.02 | 0.87 | 1.30 | +0.27 |
| High LDL cholesterol | 0.88 | 0.90 | 1.33 | 0.66 | 0.76 | -0.02 |
| Intimate partner violence | 0.80 | 0.69 | 0.73 | 1.29 | 1.18 | +0.11 |
| Drug use | 0.49 | 0.75 | 2.26 | 1.46 | 1.06 | -0.25 |
| Other environmental risks | 0.49 | 0.59 | 0.79 | 0.33 | 0.61 | -0.10 |
| Low physical activity | 0.32 | 0.42 | 0.27 | 0.33 | 0.39 | -0.10 |
| Unsafe sex | 0.17 | 0.12 | 0.39 | 0.20 | 0.52 | +0.05 |
| Unsafe water, sanitation, and handwashing | 0.09 | 0.04 | 0.13 | 0.02 | 0.61 | +0.05 |

*Table S22*. **Summary exposure value for all risk factors, *eighteen locations*, 1990 and 2023.**

| Location | SEV 1990 (95% UI) | SEV 2023 (95% UI) | 2023 as % of 1990 |
|---|---|---|---|
| Czechia | 27.59 (23.98 to 30.03) | 26.75 (23.66 to 29.11) | 96.9 |
| Bosnia and Herzegovina | 25.03 (21.86 to 27.34) | 25.81 (22.61 to 28.17) | 103.1 |
| Western Europe | 24.35 (21.27 to 26.70) | 24.94 (21.54 to 26.97) | 102.5 |
| North Macedonia | 22.81 (19.84 to 25.16) | 24.73 (21.95 to 26.85) | 108.4 |
| Slovenia | 23.26 (19.81 to 26.27) | 24.58 (22.01 to 26.63) | 105.7 |
| Serbia | 23.23 (19.56 to 25.38) | 24.15 (21.09 to 26.40) | 103.9 |
| Romania | 23.14 (20.20 to 25.26) | 24.02 (21.14 to 26.00) | 103.8 |
| Montenegro | 22.94 (19.20 to 25.12) | 23.70 (20.93 to 26.05) | 103.3 |
| Central Europe | 23.35 (20.00 to 25.26) | 23.66 (20.76 to 25.28) | 101.3 |
| Global | 24.53 (21.67 to 26.00) | 23.59 (20.66 to 24.96) | 96.2 |
| Albania | 23.01 (19.58 to 24.96) | 23.46 (20.39 to 25.78) | 102.0 |
| Hungary | 24.56 (21.36 to 26.77) | 23.46 (20.72 to 25.56) | 95.5 |
| Eastern Europe | 23.10 (19.82 to 25.11) | 23.25 (20.54 to 25.30) | 100.6 |
| Central Europe, Eastern Europe, and Central Asia | 22.79 (19.45 to 24.71) | 22.96 (20.37 to 24.77) | 100.7 |
| Poland | 22.88 (19.72 to 25.07) | 22.90 (19.49 to 24.85) | 100.1 |
| Croatia | 21.00 (17.43 to 23.58) | 22.66 (19.88 to 25.31) | 107.9 |
| Bulgaria | 20.76 (17.80 to 23.27) | 22.15 (19.15 to 24.20) | 106.7 |
| Slovakia | 22.28 (18.70 to 24.68) | 22.07 (19.23 to 24.10) | 99.1 |
